# A guide for concurrent TMS-fMRI to investigate functional brain networks

**DOI:** 10.3389/fnhum.2022.1050605

**Published:** 2022-12-15

**Authors:** Justin Riddle, Jason M. Scimeca, Mattia F. Pagnotta, Ben Inglis, Daniel Sheltraw, Chris Muse-Fisher, Mark D’Esposito

**Affiliations:** ^1^Department of Psychiatry, University of North Carolina at Chapel Hill, Chapel Hill, NC, United States; ^2^Department of Psychology, University of California, Berkeley, Berkeley, CA, United States; ^3^Helen Wills Neuroscience Institute, University of California, Berkeley, Berkeley, CA, United States; ^4^Henry H. Wheeler Jr. Brain Imaging Center, University of California, Berkeley, Berkeley, CA, United States

**Keywords:** transcranial magnetic stimulation (TMS), functional magnetic resonance imaging (fMRI), simultaneous TMS-fMRI, artifact removal, frontal-striatal circuits

## Abstract

Transcranial Magnetic Stimulation (TMS) allows for the direct activation of neurons in the human neocortex and has proven to be fundamental for causal hypothesis testing in cognitive neuroscience. By administering TMS concurrently with functional Magnetic Resonance Imaging (fMRI), the effect of cortical TMS on activity in distant cortical and subcortical structures can be quantified by varying the levels of TMS output intensity. However, TMS generates significant fluctuations in the fMRI time series, and their complex interaction warrants caution before interpreting findings. We present the methodological challenges of concurrent TMS-fMRI and a guide to minimize induced artifacts in experimental design and post-processing. Our study targeted two frontal-striatal circuits: primary motor cortex (M1) projections to the putamen and lateral prefrontal cortex (PFC) projections to the caudate in healthy human participants. We found that TMS parametrically increased the BOLD signal in the targeted region and subcortical projections as a function of stimulation intensity. Together, this work provides practical steps to overcome common challenges with concurrent TMS-fMRI and demonstrates how TMS-fMRI can be used to investigate functional brain networks.

## 1 Introduction

Transcranial magnetic stimulation (TMS) allows for direct causal manipulation of the cerebral cortex by inducing an electric field in a focal region of the brain near the scalp (Thielscher et al., 2011; Deng et al., 2013; Siebner et al., 2022). Due to its high spatial (<1 cm) and temporal resolution (<1 ms), this methodology proved essential for testing the causal role of neural oscillations (Sauseng et al., 2009; Thut et al., 2011; Riddle et al., 2019, 2020a) and the precise timing of neural activity (Pitcher et al., 2007) in specific areas of the brain. Furthermore, TMS to a brain region is now understood to alter activity within a network of structurally and functionally connected brain regions (Quentin et al., 2016; Romei et al., 2016; Hermiller et al., 2020; Ryan et al., 2022). By combining TMS with simultaneous or concurrent functional magnetic resonance imaging (fMRI), the effect of TMS on local and distant brain activity can be empirically investigated.

Magnetic resonance imaging scans rely on homogenous magnetic fields. Therefore, the extent of signal corruption derived from the simultaneous application of a strong magnetic field with TMS has long been appreciated (Bohning et al., 1999, 2003). Efforts have been made to address the complex interaction of TMS and fMRI (Bestmann et al., 2008b). However, fewer than 100 experimental studies to date have utilized concurrent TMS-fMRI (Mizutani-Tiebel et al., 2022), despite almost 20 years since its advent. The slow growth of this promising methodology is most likely due to an incomplete understanding of how an operating TMS coil impacts the MRI signal and the resulting lack of consensus regarding the most effective setup and methods for addressing unwanted artifacts (Mizutani-Tiebel et al., 2022). Several factors preclude a single, standardized approach to concurrent TMS-fMRI, including intrinsic differences in the technical characteristics of the available MRI scanners, receiver coils, and equipment to control the delivery of TMS pulses. Despite these factors, a better understanding of the common challenges and underlying principles can improve consistency in applying concurrent TMS-fMRI and the subsequent data preprocessing.

Our study provides a comprehensive, detailed investigation of the different sources of artifacts in concurrent TMS-fMRI. Building on this knowledge, we present a methodological framework for TMS during continuous fMRI, which minimizes the induced artifacts in experimental design and post-processing. First, we provide a review of hardware and practical steps for conducting a TMS-fMRI experiment. Second, we explore techniques for interleaving TMS with fMRI and summarize immediate artifacts arising from TMS during the acquisition of fMRI data. While many previously published concurrent TMS-fMRI studies interleaved TMS with a gap in fMRI acquisition, we explore TMS during continuous MRI to reduce inefficiencies in data acquisition. Third, we characterize typical artifacts that arise in post-processing with recommendations on their removal. The theoretical principles and practical steps identified here should broadly apply to other researchers who wish to conduct concurrent TMS-fMRI, regardless of technical differences in hardware or software. Finally, we conducted an experiment to demonstrate the use of these methods to target two distinct frontal cortex regions and investigated the local effect of TMS and the spread of neural activation to known striatal projections of each region.

## 2 Materials and methods

Concurrent TMS-MRI data were collected at the Henry H. Wheeler Jr. Brain Imaging Center at the University of California, Berkeley, using a Siemens 3T MAGNETOM Trio (Erlangen, Germany). TMS was delivered with the MR-compatible figure-8 Mri-B91 TMS coil produced by MagVenture (Farum, Denmark) with the MagPro X100 with MagOption running software version 7.1.1. 3D stereotaxic tracking, referred to as neuronavigation, was performed using Rogue Research’s BrainSight v2.2.11 (Montreal, Canada) with a Northern Digital Polaris Spectra infrared long-range camera (Waterloo, Ontario, Canada) and custom-made MR-compatible components. Here, we describe our experimental procedure in detail with consideration of alternatives (see Section “2.1 Equipment and procedures”), describe signal artifact sources inherent to concurrent TMS-fMRI (see Section “2.2 Signal artifacts in concurrent TMS-fMRI”), explain our artifact removal approach in post-processing (see Section “2.3 Preprocessing and artifact removal during analysis”), and present an experiment that illustrates these considerations (see Section “2.4 Experimental design”).

### 2.1 Equipment and procedures

This section details our procedure for delivering spatially and temporally precise TMS concurrent with fMRI. We will discuss the essential criteria when selecting an MR receive-coil to acquire fMRI data and techniques for positioning the TMS coil within the MR receive-coil.

#### 2.1.1 fMRI data acquisition

The MR sequence utilized for full brain coverage was a T2*-weighted single-shot echo-planar imaging (EPI) sequence with 40 slices of 3.5 × 3.5 × 3.0 mm voxels, 10% distance between slices, 2-s repetition time (TR), descending slice acquisition order, phase encoding direction anterior-to-posterior, 20 ms echo time (TE), 60° flip angle (FA), and fat presaturation. To establish a steady state with respect to spin-lattice relaxation, two dummy volumes of data were obtained at the start of each time series. The first two recorded volumes were also excluded from the analysis to further ensure spin-lattice relaxation.

Before the TMS-fMRI scans, anatomical data were collected using a Siemens 12-channel receive-only head coil using a T_1_-weighted magnetization-prepared rapid gradient-echo (MP-RAGE) sequence with 1 mm isotropic voxels, 2.3 s repetition time, 900 ms inversion time, and parallel imaging *via* GeneRalized Autocalibrating Partial Parallel Acquisition (GRAPPA) with an acceleration factor of 2. A receive coil with the capacity for rapidly acquiring detailed anatomical scans is preferred as this image is used for neuronavigation. Furthermore, since the primary use of the anatomical scan is for neuronavigation, we recommend a field-of-view sufficient to ensure that both the ears and the nose are within the 3D image because these three body parts are used for stereotaxic registration.

#### 2.1.2 MR receive-coil

**FIGURE 1:**
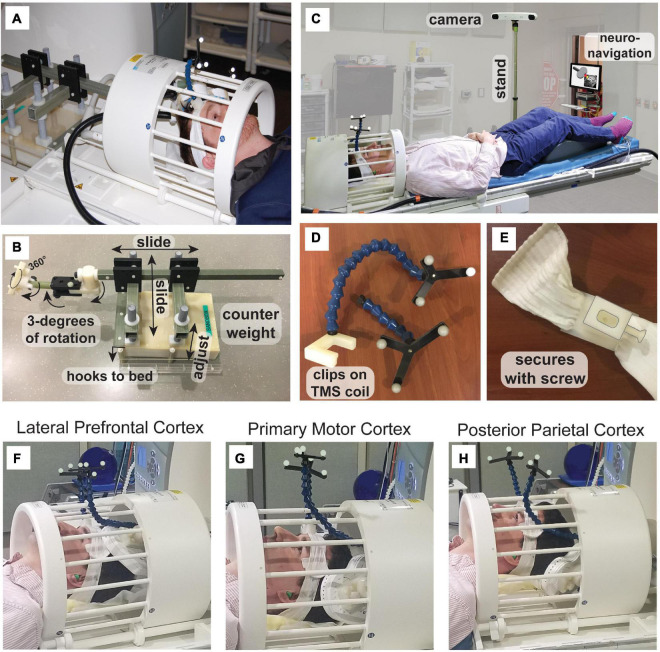
Concurrent TMS-fMRI setup. **(A)** The TMS coil enters the back of the birdcage receive coil and is supported by **(B)** a custom arm. The supporting arm can be adjusted with 3 degrees of freedom. The arm attaches to a counterweight with three translations of spatial movement along the cardinal axes of the bore. The assembly hooks to the patient bed move with the MR receive coil. **(C)** Neuronavigation can be conducted within the MRI room using a custom MR-compatible stand, such that the infrared tracking camera (not MR-compatible) can be positioned at the foot of the bed with the neuronavigation display visible from the doorway. For accurate targeting, during neuronavigation, we used **(D,E)** a custom head-tracker and coil-tracker that are detachable so that registration to anatomical image and TMS coil calibration can be conducted outside of the MRI. Example targeting of **(F)** lateral prefrontal cortex (PFC), **(G)** primary motor cortex (M1), and **(H)** posterior parietal cortex.

The choice of receive-coil to use with TMS-fMRI is critical as it directly affects what brain regions can be stimulated and imaged. Standard head volume coils, such as the Siemens 12-channel coil, have an inner diameter of approximately 25 cm and are only open at the front for head access. This does not provide enough space to move the TMS coil around the scalp of the participant, ultimately limiting the location of the brain regions that can be stimulated. To overcome this limitation, for our fMRI experiments, we used a custom-made circularly polarized, receive-only birdcage RF coil (RAPID BioMedical, Rimpar, Germany). This birdcage receive coil has a larger inner diameter (29.8 cm) than standard head volume coils (25.0 cm), and it is open at the back (Figure 1A). Hence, this receive coil allows for the TMS coil to be flexibly maneuvered around the scalp with the cable exiting the back of the birdcage coil. Since the effect of TMS on brain activity is known to be network-wide as well as local to the site of TMS, quantifying its impact across the entire cortex and subcortex is essential to understanding the full effects of TMS. Thus, we chose to pursue a method for acquiring whole-brain fMRI. By contrast, some groups have chosen to image local areas of the brain with flexible coils that cover a portion of the skull resulting in partial spatial coverage (de Weijer et al., 2014; Navarro de Lara et al., 2015, 2017). We acknowledge that practical constraints can limit the decision for MR receive-coil, and some groups have successfully acquired concurrent TMS-fMRI by removing the top half of a standard receive coil or using a body coil (Bestmann et al., 2010).

In our setup, the TMS coil is attached to a custom-made support arm (Figures 1A, B). This custom TMS arm is made of plastic with a counterweight that consists of an MR-compatible plastic box with sand inside (weight 5.7–5.8 kg; dimensions: 25.1 cm × 35.2 cm × 7.7 cm). The support system attaches to the patient bed runners, so it slides into the magnet’s bore from the rear. Hooks secure the TMS coil arm support to the patient bed. The arm includes 6 degrees of freedom: three translations along the magnet cardinal axes, plus a TMS coil mount that provides three separate rotation axes (Figure 1B). With dorsal, lateral, and anterior cortical targets, the TMS coil can be easily positioned around the top, side, and front of the head. Regardless of the position of the TMS coil with respect to the head, researchers should ensure that there is no pressure on the head of the participant, as this will be increasingly uncomfortable over the course of the experimental session. We recommend using large cushions with high resistance and smaller cushions with less resistance to fill in smaller sections. These cushions are typical MR accessories used to pack the head into the receiver coil to reduce movement. Here, these cushions take on the additional role of stabilizing the scalp with respect to the TMS coil.

To target the site of interest accurately, we used neuronavigation within the MRI room (Figure 1C). The magnetic stand of the long-range infrared camera was replaced with a custom stand constructed of plastic, allowing the camera to be used at the foot of the patient bed. An iMac computer running BrainSight was kept at the doorway to the MRI room and secured to the wall using a plastic chain to avoid it being taken in the high magnetic field. We custom-designed and 3D-printed head and coil trackers using the dimensions of the head and coil trackers from BrainSight for use in the MRI (Figures 1D, E). The head tracker is attached to a headband that the participant wears. Crucially, the tracker can be detached from the base and reattached within the MR receive coil. Similarly, the TMS coil tracker can be removed from the TMS coil and reattached within the MR coil. All items are marked as MR-compatible when applicable and careful screening was applied to all users and participants. A custom-built mirror system was mounted to the MR coil to provide the participant visibility to the back-projected screen (AVOTEC, Stuart, FL, USA), and this included an optional eye-tracking mount (AVOTEC) that was not used in this study. Our procedure for neuronavigation in the MR environment involved three stages (Table 1) and took place after calculating the region of interest within a previously acquired high-resolution anatomical image.

**TABLE 1:** Neuronavigation in the MRI room for precision targeting.

Stage	Participant	Steps	Rationale	Alternatives
1. Approximate targeting	Seated in the MRI room on an MR-compatible gurney	Participant wears the head tracker and is registered to neuronavigation software. Then, the entry location is marked on the scalp with a washable marker.	If precision targeting in Stages 3 and 4 is attempted, then the desired TMS coil location may require you to restart at Stage 3. Stages 1 and 2 make it less likely that patient registration and coil calibration will need to be repeated.	If targeting the motor hotspot, then this location can be directly marked. If the target site is highly canonical across participants, then this step might be skipped.
2. Final adjustment of flexible head and coil trackers	Laying on the MR patient bed, not moved into the bore.	TMS coil is moved into approximate position on the scalp. Flexible head and coil trackers are attached and adjusted to protrude from the receive coil to maximize range of potential movement. Ensure that the head and coil trackers are visible to the infrared camera. Support cushions are added.	The TMS coil calibration and subject registration in Stage 3 might result in the flexible trackers being bent into a bar of the receive-coil in Stage 4 such that Stages 3 and 4 must be repeated.	If using flex-coils, then Stages 1 and 2 can be skipped.
3. Final registration and TMS coil calibration	Laying on MR-compatible gurney in the MRI room	TMS coil calibration with coil tracker from Stage 2. Patient registration with head tracker from Stage 2.	Participant must be supine because the head tracker will be angled toward the feet.	With high confidence in the final coil to head placement, Stage 3 could theoretically be the first step.
4. Precision targeting	Laying on the MR patient bed not in the bore.	TMS coil fixed to support arm. Head and coil trackers attached and adjusted for precision targeting (<5 mm and <5°). Participant wears ear plugs. Pack the receive coil with cushions.	The TMS coil will already be close to the final target. Stage 2 created room for fine adjustments in this stage.	If investigators do not utilize precision targeting, then techniques for calculating the coil target from high-resolution MR images should be used.
5. Move into bore	Laying on the MR patient bed in the bore.	Trackers are removed. The patient bed is moved into the bore. Neuronavigation is removed from the MRI room.	The support arm is hooked to the patient bed and the cushioning will ensuring that the position is maintained.	Using this technique, the location of the TMS coil at the end of the session can be confirmed.

Using a five-stage process, the location of the TMS coil was ensured to be positioned over the target with less than 5 mm of error in translation and less than a 5° error in angle.

An advantage of using a birdcage coil is that it allows for TMS of most of the dorsal and anterior regions of the cerebral cortex, e.g., lateral prefrontal cortex (PFC; Figure 1F) or primary motor cortex (M1; Figure 1G). For a posterior site like inferior intraparietal sulcus, the head should be lifted with cushions around the neck in such a way that the TMS coil can be positioned under the head (Figure 1H). In testing, we were able to target the majority of sites on the scalp that are traditionally targeted with TMS with the exception of cerebellum and the ventromedial prefrontal cortex (VMPFC). The participant lays supine and, thus, with TMS to cerebellum the participant would need to rest their head on the TMS coil which is uncomfortable and has the potential to damage the TMS coil from the weight of the head. TMS to primary visual cortex is theoretically possible because the neck can be supported with cushions leaving the full scalp exposed. TMS to VMPFC is so far anterior that the space between the forehead and MR receive coil is limiting, although if the participant does not need to see the screen or if a system of mirrors can be constructed, then perhaps TMS to VMPFC would be in principle feasible using a similar setup to the one presented here.

### 2.2 Signal artifacts in concurrent TMS-fMRI

Transcranial magnetic stimulation involves the production of brief, intense magnetic fields over the scalp of the participant. When performed inside an MRI scanner, there are several ways in which the TMS and MRI hardware can interact (Bestmann et al., 2003a; Siebner et al., 2009; Weiskopf et al., 2009). Most obviously, the TMS pulses of intense magnetic field interact with the nuclear magnetization and perturb the MR image formation. The precise consequences of the interaction depend on factors such as the magnetization at the time of the TMS pulse and the magnitude and spatial heterogeneity of the TMS magnetic field. Additionally, artifacts can arise even when the TMS coil is not actively sending a pulse. For example, the TMS coil, which is made of copper wire, has a magnetic susceptibility that is different from the foam pillows or other items that are typically placed around the head of a participant for comfort or support. The presence of the coil can lead to local signal dephasing from magnetic susceptibility gradients, as well as shading of the transmission RF field. MR pulse sequences and image processing tools were not developed with consideration of these factors. Finally, there may be inefficiencies in the TMS control electronics that are unavoidable within feasible constraints which lead to unintentional weak magnetic fields in the TMS coil between pulses.

In Table 2, we list artifacts that can occur in fMRI data when a TMS coil is within the bore of a MRI scanner, or when a TMS coil delivers pulses during fMRI acquisition. The artifacts listed in Table 2 are not intended to be all inclusive and are categorized by their physical origin. We do not list the possible severity of these artifacts since they are strongly influenced by the particular hardware choices and experimental set up (e.g., the position of the TMS coil relative to the head of the participant). We have given approximate temporal scales for each potential artifact, but these estimations will also be influenced by these factors. The contents of Table 2 are explored in more detail in the Supplementary material.

**TABLE 2:** MRI artifacts that may arise during data acquisition due to interactions with concurrent TMS.

Origin	Artifact description	Temporal dynamic	Effect on MRI signal
TMS coil	Magnetic susceptibility	Persistent	Reduced SNR under TMS coil
	RF shading	Persistent	Reduced SNR under TMS coil
	Vibration	ms	Reduced SNR under TMS coil
	Thermal drift	min	Changing signal intensity under TMS coil
	RF noise introduced through TMS cable	Persistent	Global reduction of SNR in all MR images
	RF spikes from friction inside TMS coil	μs-ms	RF interference stripes, reduced SNR
TMS power source	Leakage current (Weiskopf et al., 2009)	Tens of ms	Reduced SNR under TMS coil
	Asymmetric biphasic pulse	Variable	Imparts phase to transverse magnetization
TMS to MRI hardware coupling	Eddy currents in MRI hardware	μs-sec	Residual after TMS pulse acts as spoiler gradient
	Receive coil to TMS coil inductive coupling	Permanent	Reduces quality factor of Rx coil: shift from subject noise to TMS coil/cable dominance
	Ring-down in receive coil decoupling electronics	μs-ms	May cause signal reception issues, even RF coil failure.
	Transmitter coil to TMS coil inductive coupling	Permanent	Reduces quality factor of Tx coil; shift from sample noise to TMS coil/cable dominance
	Ring-down in transmit coil decoupling electronics	μs-ms	May cause Tx/Rx decoupling issues
TMS to EPI sequence coupling	Perturbation of M_0_ *via* an effective precessional field	ms-sec	Erroneous image contrast (esp. T1), reduced SNR, and possibly image artifacts
	Errors in slice selection	Multiple TRs	Corrupted current slice and possibly corrupted later slices
	Errors in fat suppression	Multiple TRs	Corrupted current slice, possibly corrupted later slices.
	Corrupted k-space	Single slice	Unusable current slice (or multiple slices if using simultaneous multi-slice acquisition)
	Flow/diffusion weighting	<1 s	Erroneous contrast

SNR, signal-to-noise ratio; RF, radio frequency; TR, time of repetition and refers to a single 3D volume of data; EPI, echo planar imaging and is the commonly used sequence for functional MRI.

Our detailed investigations of the artifacts in concurrent TMS-fMRI suggest that it is difficult to isolate residual artifacts from leakage current (Weiskopf et al., 2009), vibration, and ring-down in the receiver coil. All three effects decay in the milliseconds following a TMS pulse. We suspect that leakage current remains the dominant post-TMS artifact, even though our MagVenture system was installed with an upgrade that reduces the leakage current to some extent by using a relay-diode combination inserted in the TMS circuit that shorts the leakage current. While the intensity of leakage current magnetic fields is several orders of magnitude smaller than the TMS pulses and is of minor consequence from the perspective of stimulating the brain, the spurious leakage current magnetic field is sufficiently large to perturb magnetization during EPI. Given the subtle changes in the BOLD signal often measured by fMRI experiments, these leakage currents can produce significant local image disturbances near the TMS coil, particularly in the 10–20 ms after a TMS pulse as the capacitor recharges. We take this delay into account for the specific timing used in our experiments and note that this delay also allows recovery of any vibration of the TMS coil, electric field discharges within the TMS coil, and recovery of ring-down in the receiver coil electronics.

With the low temporal resolution of MRI, the artifacts produced by leakage currents from the TMS power supply, eddy currents in MRI scanner components, and vibrations of the TMS coil in response to a pulse are more or less indistinguishable from each other in their effects upon the MR images. Each source of artifact will generate signal dropout surrounding the 3D location of the TMS coil. The spatially localized dropout returns to baseline further from the source of the artifact. The time constants can also be expected to overlap.

Considering these artifacts, it is crucial to deliver TMS with high temporal precision to avoid stimulating during critical events in the EPI sequence. For example, TMS delivered during the RF excitation stages of the EPI sequences produces a long-lasting (for many volumes) and prominent disruption of the MRI signal and should be avoided. We developed a custom microcontroller-based synchronization system to establish precision in TMS pulse delivery relative to the MRI sequence. A Hercules RM42x LaunchPad microcontroller board (Texas Instruments, Dallas, TX, USA) was used to control the synchronization. The microcontroller board was programmed to issue a hardware interrupt upon receiving a synchronization signal from the scanner. For convenience, we utilized the TTL pulse emitted at the start of each TR period. An interrupt service routine then schedules a signal sent to the trigger port of the TMS unit at a user-defined time relative to the start of a volume acquisition. The trigger port of the TMS unit is also hardware interrupt-controlled and, upon receiving the signal from the microcontroller board, issues a pulse. Since the synchronization signal from the scanner is sent at a fixed time within the EPI sequence for each image volume, and since all signals were detected through hardware interrupts, we were able to obtain completely deterministic synchronization of TMS to EPI events, with a guaranteed timing precision of less than one microsecond. Such precision is required for timing the placement of TMS pulses relative to the MRI sequence events during continuous MRI.

**FIGURE 2:**
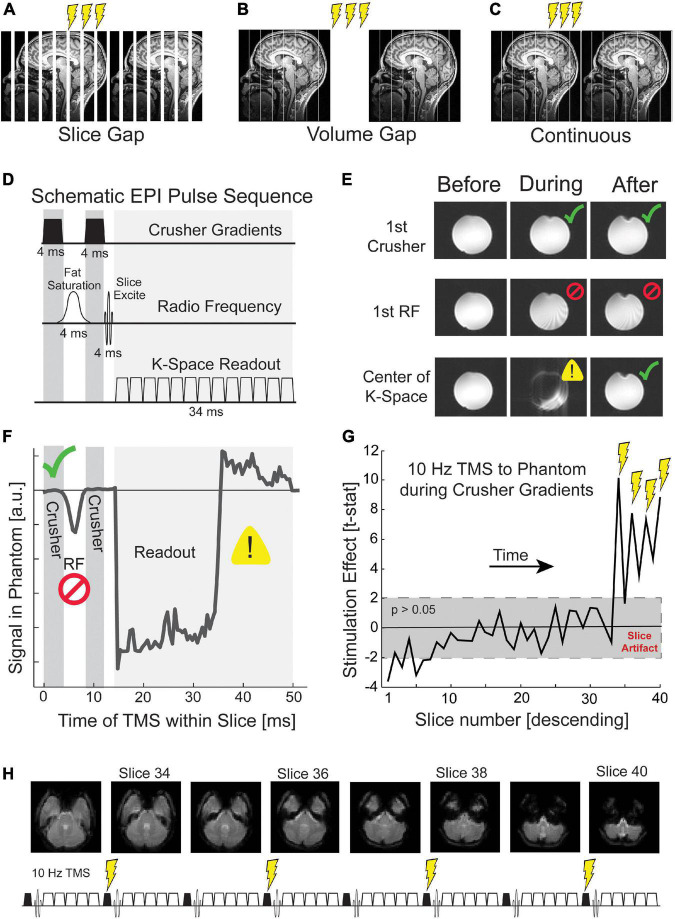
Targeting crusher gradients with TMS in continuous fMRI. TMS pulses during fMRI can be delivered **(A)** in imposed gaps between slices, **(B)** in imposed gaps between volumes, **(C)** or continuous with fMRI acquisition. **(D)** An MRI slice comprises three epochs: crusher gradients, radio frequency excitation, and k-space readout. The crusher and readout gradients are depicted symbolically. Actual gradients are administered on all three gradient channels and have differing timing depending on the intended use. **(E)** TMS that occurs during each of these three epochs generates different artifacts. *Before* refers to the volume (i.e., set of EPI slices) acquired before TMS, *during* to the volume of TMS, and *after* to the volume following TMS. TMS during the first crusher gradient shows minimal distortion (green check; top row), TMS during the first RF pulse causes persistent disruption (middle row; red cross), and TMS during k-space readout disrupts only the current slice (bottom row; yellow warning). **(F)** TMS incremented throughout the acquisition of a slice reveals unique signal disruption such that the temporal relationships between the three epochs of slice acquisition can be visualized. Further details are given in the Supplementary material. **(G)** TMS during the first crusher gradient produces slice artifacts that are not visible to the naked eye but are detectable statistically. The gray box depicts a *t*-test with a *p*-value greater than 0.05. **(H)** Our continuous TMS-fMRI paradigm delivered four pulses of TMS during the crusher gradients of slices 34, 36, 38, and 40. These slices correspond to the acquisition of the cerebellum and temporal poles.

Some previously published TMS-fMRI experiments added a delay between every slice of a multi-slice EPI volume (slice gap; Figure 2A) (Moisa et al., 2009; Hermiller et al., 2020). In this framework, which we shall call slice interleaved TMS-fMRI, single pulses or trains of TMS can be delivered at intervals compatible with the gap timing, i.e., one pulse or burst per an integer number of EPI slices. However, this approach decreases the quantity of fMRI data that can be collected in a given period. Alternatively, a delay in scanning can be added between each volume of MR acquisition (volume gap; Figure 2B) (Bohning et al., 1998; Nahas et al., 2001; Ruff et al., 2006; Oathes et al., 2021), an approach we shall refer to as volume interleaving. The delay must be included for every volume of the EPI sequence whether or not TMS pulses are applied, rather than before specific volumes only, to avoid perturbing the T_1_ steady state of the MRI signal. This approach also reduces the amount of fMRI data collected per unit of time. We present here a timing paradigm that applies TMS pulses during certain “safe” events in the continuous EPI time series (Figure 2C), which eliminates the need for a gapped EPI acquisition and allows fMRI data collection at the usual rate. The safe EPI acquisition events identified as the candidate timing for continuous TMS-fMRI were the crusher gradients.

Crusher gradients are magnetic field episodes intended to scramble the phase of unwanted magnetization. There are two principal objectives. Crusher gradients immediately following the fat saturation RF pulse are designed to eliminate coherent fat signal and minimize fat signal at the time of k-space read-out. Crusher gradients at the end of a slice acquisition, following the 2D k-space read-out, are designed to ensure that signal from the prior slice is fully destroyed before exciting and detecting the subsequent slice. The TMS coil produces a biphasic pulsed magnetic field that is asymmetrical in time such that the second phase is marginally weaker than the first phase. The biphasic TMS pulse acts as a crusher gradient six orders of magnitude greater than the approximately 40 millitesla crusher gradients imposed by the scanner. Ironically, improved spoiling of unwanted MR signals using TMS pulses could lead to a situation in which the BOLD signal acquired during TMS-fMRI has improved signal-to-noise ratio relative to the BOLD signal without TMS, e.g., residual scalp fat signal, which tends to create pronounced N/2 ghosts, might be reduced with TMS. The technical difficulty imposed in targeting the crusher gradients is that these gradients are brief, around 3 ms in the standard Siemens implementation. To reliably target the crusher gradient requires sub-millisecond precision timing of TMS. The crusher gradients each precede an RF pulse, which is the most disruptive time to apply TMS. Thus, targeting the crusher periods also requires that we pay particular attention to any TMS artifacts that remain once a TMS pulse has been applied.

Our timing solution for continuous TMS-fMRI acknowledges potential sources of artifact. We considered three epochs during EPI for which TMS has significantly different effects (Bestmann et al., 2008b): (1) the RF pulses for fat saturation and slice excitation, (2) gradient episodes sampling a k-space plane, and (3) crusher gradients that reset the MR signal between slices or spoil coherence produced by the fat suppression pulse (Figure 2D). To investigate the impact of TMS on each of these EPI phases, we delivered single-pulse TMS to a Functional Biomedical Informatics Research Network (FBIRN) standard gel phantom (Friedman and Glover, 2006; Greve et al., 2011). TMS pulses were delivered every three volumes, and the precise time of TMS was during the acquisition of a slice near the center of the phantom. With a two-second TR and 40 slices, each slice was collected for 50 ms. Thus, 51 pulses were delivered over the course of the EPI sequence, where each pulse was delivered at a different 1 ms interval of slice acquisition. The mean signal within the temporally targeted MR slice was calculated for each TMS pulse and compared to the mean signal for the previous volume. This analysis was designed to quantitatively illustrate the impact of TMS on the MR signal for each epoch of slice acquisition.

### 2.3 Preprocessing and artifact removal during analysis

Preprocessing steps are required to clean the fMRI data before statistical analysis. The eddy/leakage currents or mechanical vibrations within the TMS coil can perturb the local MR signal for multiple volumes beyond TMS, however, these coil artifacts are not addressed in most published studies of concurrent TMS-fMRI. Without explicit modeling, false positive changes due to local distortion cannot be ruled out. Due to artifacts under the TMS coil, it is difficult to validate the efficacy of TMS in this site leading to studies to focus on distal effects of TMS. Whether or not TMS evokes neural activity in the site being stimulated as measured by the BOLD signal is currently debated, with a recent review paper claiming the BOLD signal does not increase with TMS (Rafiei and Rahnev, 2022) unless M1 is targeted (Bohning et al., 1999, 2000, 2003; Bestmann et al., 2003b). Our methodology characterizes and regresses out fMRI data associated with the coil artifact *via* Independent Component Analysis (ICA), and we used a standardized means of verifying that the BOLD signal in a region is not driven by a false positive.

Even though the effect of TMS when delivered during the first crusher gradient is not visible to the naked eye, we replaced each slice acquired during TMS with the average of the same slice in the preceding and subsequent volume. While interpolation loses some temporal resolution, the net gain in precision from not including a volume delay or slice delay is preserved. Furthermore, the slices that are corrupted by the timing of TMS can be selected as regions of non-interest. After slice interpolation, we applied FSL’s MELODIC algorithm (Jenkinson et al., 2012), which performs an ICA that decomposes the signal into various signal sources or independent components (ICs). Next, we ran a general linear model (GLM) that predicted a near-instantaneous signal drop (dependent variable), typical of TMS artifacts using the time series of each IC as the independent variables. This artifact time series was modeled as a zero for every volume without TMS and a negative one for each volume with a train of TMS. After identifying ICs with a high explained variance from the predicted artifact time course (greater than 10%), we manually inspected the spatial correlation of each component and rejected those associated with TMS coil discharge. The threshold of 10% was selected based on thresholding convention from other techniques, such as factor analysis (Yong and Pearce, 2013), and the use of a lower threshold would require more manual inspection. In some participants, we also discovered Ics with a spatial correlation localized to the slices collected during TMS. After rejecting Ics localized to TMS coil discharge, preprocessing was carried out in the Statistical Parametric Mapping 12 (SPM12) Toolbox for Matlab unless otherwise noted. Data were despiked (AFNI), manually reoriented to the anterior commissure, slice time corrected, realigned to the mean functional image using rigid body rotation, coregistered to the anatomical image, and smoothed with a 4-millimeter full width at half maximum Gaussian kernel.

### 2.4 Experimental design

Previous evidence from positron emission tomography (PET) demonstrated that TMS to M1 and PFC resulted in the extracellular release of dopamine in the posterior putamen and dorsal caudate, respectively (Strafella et al., 2001, 2003). These findings are consistent with structural connectivity studies that find an anterior-to-posterior gradient mirrored in the frontal cortex and striatum such that PFC preferentially projects to the anterior caudate and M1 preferentially projects to posterior putamen (Draganski et al., 2008; Jarbo and Verstynen, 2015; Gordon et al., 2022). We conducted an experiment to test the effectiveness of our concurrent TMS-fMRI procedure and to investigate whether we found evidence of increased local activity under the TMS coil and activation in the *a priori* striatal regions of interest. After developing a protocol for continuous fMRI acquisition with concurrent trains of 10 Hz repetitive TMS (rTMS), we targeted either the right hand-knob of M1 or the right anterior, middle frontal gyrus of PFC in two groups of participants. We hypothesized that TMS during the eyes-open resting state would activate the putamen when delivered to the right M1 and the head of the caudate when delivered to the right anterior, middle frontal gyrus. While these hypothesized projections are consistently found across studies, other groups found more diffused projections from M1 and lateral PFC (Draganski et al., 2008; Averbeck et al., 2014). Thus, we evaluated the specificity of TMS by investigating its impact on each region of the dorsal striatum.

The study was approved by the Committee for the Protection of Human Subjects at the University of California, Berkeley. Fifteen participants (ages 18–29, mean 21.6, standard deviation 2.5) participated in the study, which consisted of two fMRI sessions. In the first session, we acquired an individual anatomical whole-brain image and 10 min of eyes-open resting state fMRI data while participants were instructed to fixate on a centrally presented fixation cross. These data were collected with a 12-channel MR receive-coil. The second session consisted of a concurrent TMS-fMRI experiment and differed between participants in the location of TMS: either right M1 (8 subjects) or PFC (7 subjects).

#### 2.4.1 Repetitive TMS parameters

Each pulse of TMS was delivered during the first crusher gradient of slice acquisition (the crusher that eliminates transverse magnetization left over from the prior slice) as this time was shown to generate the least artifact. The EPI timing parameters were set such that TMS could be delivered in increments of 50 ms, which enabled bursts of TMS to be delivered at 10 Hz, i.e., every other slice. As this was the frequency of TMS used by Strafella et al. (2001, 2003) and by many of the early studies of concurrent TMS-fMRI (Ruff et al., 2006), these results could be compared to previous work. In the concurrent TMS-fMRI session, TMS was delivered at four different intensities: 40, 60, 80, or 100% of resting motor threshold. The order of TMS intensity was randomized and counterbalanced within each block. In each run, participants received 48 bursts of 4 biphasic pulses of TMS at 10 Hz. The inter-burst interval was randomized and counterbalanced to be either 6, 8, 10, or 12 s. The participants that received TMS to M1 underwent four runs of TMS-fMRI, whereas participants that received TMS to PFC received one run of TMS-fMRI. The difference in the number of runs per site was not related to the goals of this study.

Outside the scanner, single-pulse TMS was delivered over the right M1 using neuronavigation to the hand knob. TMS intensity was increased, and the coil was adjusted until TMS reliably evoked a visible twitch in the first dorsal interosseous muscle of the left hand on 5 out of 10 pulses, i.e., the resting motor threshold. When the resting motor threshold was found, we marked the location on the anatomical image of the participant. This region of interest was used for TMS targeting in concurrent TMS-fMRI and for region of interest analysis.

We ran a functional connectivity analysis of the baseline resting-state scan without TMS to define the anterior middle frontal gyrus of the PFC as a TMS target and to be used in analysis. Resting-state functional connectivity analysis was preprocessed as described in Section “2.3 Preprocessing and artifact removal during analysis” but without the TMS-specific cleaning steps. A GLM was run with regressors for motion (three spatial translations), average white matter signal, and average cerebral spinal fluid signal. The residual images from the GLM were used for the connectivity analysis. Peak functional connectivity with the right superior precentral sulcus (sPCS) was used to define anterior, middle frontal gyrus, and superior intraparietal sulcus (IPS) because the sPCS can be reliably identified by anatomical landmarks: at the intersection of the precentral sulcus and the superior frontal sulcus. A seed-based connectivity analysis was also run in IPS and PFC to confirm that these regions formed an interconnected functional network. The group average coordinate in the Montreal Neurological Institute (MNI) space was (Brett et al., 2002; Blankenburg et al., 2008; Ryan et al., 2022) for M1 and (Bohning et al., 2000; Strafella et al., 2001; Draganski et al., 2008) for PFC. This method was established to test hypotheses outside the scope of the current experiment.

#### 2.4.2 Statistical analysis

Our first-level GLM had four regressors of interest and eight nuisance regressors. Each train of repetitive TMS was modeled as a boxcar with a duration equal to the duration of the TMS burst and convolved with the canonical hemodynamic response function (HRF). The four regressors of interest were the four intensities of TMS (40, 60, 80, and 100% of resting motor threshold). Of the eight nuisance regressors, six modeled motion regressors generated by SPM12 realignment algorithm for three translations and three rotations. The other two nuisance regressors were the mean signal of global white matter and cerebral spinal fluid. Tissue estimations were calculated using the segmentation algorithm with default parameters in SPM12. We defined our regions of interest (ROI) in posterior putamen and dorsal caudate based on two previous studies that applied repetitive TMS to M1 and PFC, respectively, and found the maximum amount of extracellular dopamine release (as measured by a reduction in [^11^C]raclopide signal in PET scanning) (Strafella et al., 2001, 2003). A 6 mm diameter ROI was defined with the center of mass at (Jenkinson et al., 2012; Riddle et al., 2019; Siebner et al., 2022) for putamen and (Bohning et al., 1999; Bestmann et al., 2008b; Quentin et al., 2016) for caudate using the MarsBaR toolbox (Brett et al., 2002). These regions were defined in the Montreal Neurological Institute (MNI) normalized space.

After the GLM was run for each participant, the contrast images were normalized into MNI space for group-level analysis. We extracted the mean evoked BOLD activity from the univariate analysis for all ROIs: M1, PFC, caudate, and putamen. The contrast images of interest were the four TMS intensities and a contrast image for the parametric effect of TMS intensity. We ran a two-way analysis of variance (ANOVA) with a between-participants factor for TMS to M1 or PFC and a within-participant factor for the BOLD signal in the ROIs: either M1 and PFC or putamen and caudate.

A finite impulse response (FIR) model was run to confirm that scanner artifacts did not drive our BOLD effects. The FIR model was chosen to capture the 12 s following TMS in six gamma functions, each lasting the duration of a single TR of 2 s. Therefore, the GLM for the FIR model has 24 regressors of interest, six for each of the four TMS intensities, and the eight nuisance regressors.

## 3 Results

This section summarizes the findings from developing a novel procedure for concurrent TMS-fMRI. While collecting pilot data, we observed that the coil output of TMS was systematically greater within the MRI (see Section “3.1 TMS intensity at the isocenter of the MRI”). By timing the delivery of TMS with microsecond precision, the induced artifact from TMS at each epoch of slice acquisition can be estimated, and the optimal time of TMS can be determined (see Section “3.2 Temporally targeting the crusher gradients”). Our preprocessing approach removed signal artifacts reflecting slice distortion temporally locked to TMS delivery and reflecting artifacts from within the TMS coil itself. Our statistical analysis demonstrated that these artifacts could be removed (see Section “3.3 Independent component analysis removes TMS coil artifacts”). Finally, we present our findings from an experiment illustrating that our approach delivering TMS during continuous fMRI revealed local and distal effects of TMS on neural activity (see Section “3.4 Activation of frontostriatal loops from TMS was hierarchical”).

### 3.1 TMS intensity at the isocenter of the MRI

During early piloting, we observed that the participant’s subjective experience of the intensity of TMS was greater while inside the MRI bore than outside of the bore. To test for a systematic difference in the TMS-induced electric field, we recorded the electromotive force (emf) due to a TMS pulse by using a probe (MagVenture MagProbe) connected to an oscilloscope while at the isocenter of the MRI scanner and in the fringe field at the location where the resting motor threshold of the participants was determined. This location was near the entrance to the MRI room, approximately 3 m from the entrance to the MRI bore. The probe was attached to the center of the TMS coil, and emf was recorded in both locations. We found a systematic difference such that TMS within the isocenter of the 3T magnet produced about a two-microvolt increase in intensity relative to the fringe field location. This difference corresponds to roughly 5% of coil output for the MagVenture system. Thus, we consider the intended TMS intensity to be 5% lower within the scanner (Yau et al., 2014).

### 3.2 Temporally targeting the crusher gradients

Transcranial magnetic stimulation was delivered during every millisecond of MRI slice acquisition, and the impact on the mean signal in the acquired slice vs. the following volume was calculated (Figures 2E, F). TMS during RF excitation pulses produced artifacts in the current volume but also corrupted the MR signal for multiple subsequent volumes. TMS pulses applied during the k-space readout irretrievably corrupted that slice but did not impact subsequent slices. This is not a corrupting signal superimposed on the genuine measurement but a fundamental disruption of the signal readout; and thus, there is no linear regressor that can recover the underlying image. If it is necessary to apply TMS pulses during a k-space readout period, we recommend a linear interpolation with the neighboring volumes for that slice. Interpolation may induce its own artifact if the participant moves significantly in the volume before or after TMS. If the interpolated slice is an average of data collected from different parts of the brain due to motion, this will induce a spatial and temporal smoothing effect.

In certain experimental designs, such as high-frequency rhythmic TMS that exceeds the MRI slice acquisition rate, TMS during read-out gradients might be unavoidable. In this instance, we recommend timing the TMS to consistently occur during the acquisition of slices that are of less interest to the particular research question. For example, if the experiment does not require analysis of the BOLD signal in the cerebellum and the MR acquisition includes slices in the cerebellum, then TMS pulses can be delivered during MR acquisition within those cerebellar slices. After interpolation of the corrupted slice, there will be limited disruption of subsequent volumes.

After examining the effects of TMS throughout the MRI slice acquisition, we identified the crusher gradients as the optimal time to deliver TMS. TMS during the crusher gradients did not produce any visible distortion in the resulting image of the phantom (top row of Figure 2E) or a discernible change in the mean signal of the image (Figure 2F). To determine if this qualitative observation was statistically significant, we collected a run of concurrent TMS-fMRI with four pulses delivered at 10 Hz during the first crusher gradient of slices 34, 36, 38, and 40. Across 48 TMS trains, we calculated the mean difference in signal between each slice in the volume collected during TMS relative to the volume collected after TMS (Figure 2G). A *t*-test was run for each slice, and a significance threshold of 0.05 was set. We found that TMS produced a significant difference in signal that was maximal for the four slices that were acquired during TMS [*t*(47) > 2.01; *p* < 0.05], as well as the statistically significant residual impact of TMS on the slice acquired after each volume (35, 37, 39, and the 1st, 3rd and 5th slice of the next volume). These findings suggest the presence of leakage current in the equipment that was only a fraction of the impact of slice acquisition during TMS. Due to this finding, we temporally delivered TMS while acquiring slices from the cerebellum and interpolated data from each slice acquired during TMS and those acquired immediately after TMS (Figure 2H).

### 3.3 Independent component analysis removes TMS coil artifacts

**FIGURE 3:**
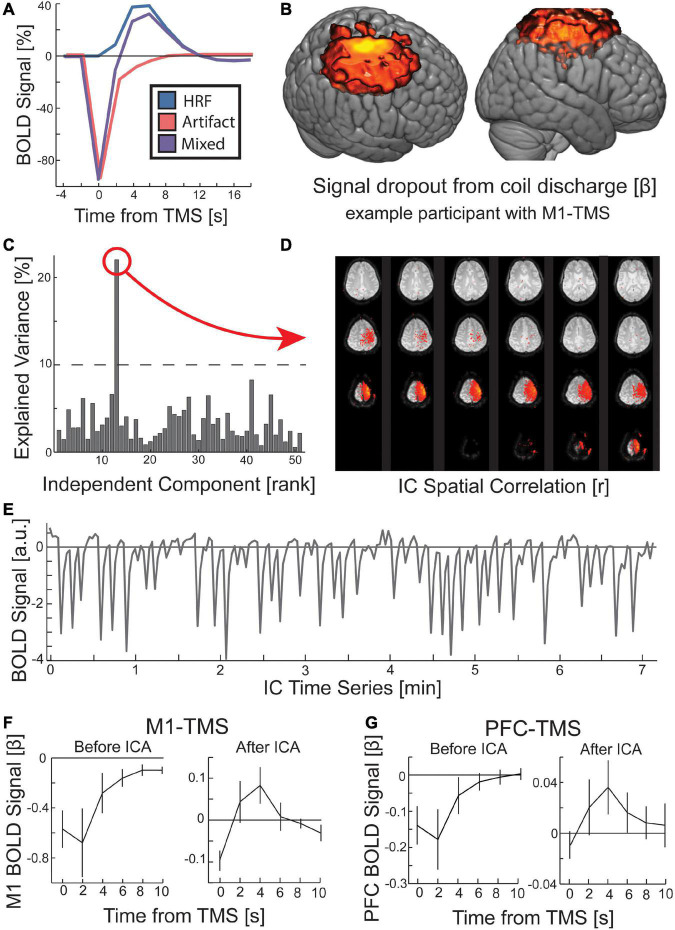
Procedure for removing the local artifact of TMS coil discharge in the preprocessing of fMRI data. **(A)** The measured BOLD activity following TMS is a mixed signal (purple) of an immediate signal drop around the TMS coil (red) and the evoked hemodynamic response function (HRF; blue). **(B)** When the canonical HRF was replaced with an inverted gamma function, a GLM for all TMS conditions revealed a significant (*p* < 0.00001; k > 1000) signal dropout at the location targeted by TMS and rising out of the scalp into the location of the TMS coil. While displayed with hot colors, this activity reflects a signal drop centered on the time of TMS. **(C)** ICA identified the TMS coil discharge artifact. GLM identified IC time course that correlated with the predicted coil artifact (threshold at 10% explained variance). The **(D)** spatial correlation and **(E)** time course of a TMS coil artifact IC are depicted. **(F,G)** Across all participants, the TMS coil artifact was present prior to ICA, but the HRF was visible after ICA rejection. Error bars are SEM.

Transcranial magnetic stimulation pulse artifacts can perturb the local MR signal for multiple TRs beyond TMS. To model and remove all TMS coil artifacts driven by eddy/leakage currents or mechanical vibrations, we first used a GLM-based approach to identify such artifacts, and then we decomposed the data into ICs and regressed out those identified as TMS coil discharge artifacts. Because the drop in signal around the coil occurred at the time of TMS, modeling a canonical HRF at the time of TMS will result in a false positive. As the signal drop returns to baseline, the increase in signal will mimic the rising edge of the HRF (Figure 3A), and standard fMRI analysis pipelines will mistake this pattern as a BOLD response. When a GLM was run on data with prominent TMS coil discharge artifacts that treated every TMS pulse as an event convolved with the canonical HRF, the coil artifact was erroneously identified as a significant BOLD activation. When an inverted gamma function was instead centered around the time of the TMS train to capture the signal drop, the resulting contrast map (Figure 3B) showed a pronounced artifact at the site of TMS extending into the space above the skull in the location of the TMS coil. This artifact reflects a decrease in signal at the time of TMS. A FIR function model revealed that this TMS coil discharge artifact tracked with the intensity of the TMS and lasted for more than two volumes post-TMS.

To identify data with TMS coil discharge artifacts, we applied FSL’s MELODIC algorithm to decompose the data into ICs, and then we ran a GLM to predict a near-instantaneous signal drop in the time course of each IC, obtained from data decomposition (see Section “2 Materials and methods”). The explained variance in the time course of the IC by the canonical artifact shape was sufficient for identifying data with artifacts (Figure 3C). The ICs with a high explained variance from the predicted artifact time course were manually rejected by inspecting their spatial correlation (Figure 3D). Using this method, we rejected ICs from the data associated with the TMS coil discharge artifact. Of note, the time course for these artifacts reveals a persistent effect (Figure 3E). In the two sites of interest under the TMS coil, M1 and PFC, the artifact comprises a sharp drop in the BOLD signal that returns to baseline over four volumes, reaching baseline at the 5th volume post-TMS (Figures 3F, G). Since our MR sequence used a TR of 2 s, the coil discharge artifact in our data was present for 8 s. After removal of the ICs corresponding to the TMS coil discharge artifact, the canonical HRF pattern is visible in the site targeted by TMS (Figures 3F, G).

In addition, our analysis of the mean slice signal when targeting the crusher gradients revealed that the slices acquired during TMS were corrupted, although this signal distortion was not perceptible *via* visual inspection. Thus, we interpolated over these slices and their subsequent slice. However, manual inspection of the ICA results revealed that some of the ICs showed spatial correlation with particular slices that were acquired at or near the time of TMS. These ICs were also removed from the data. Experimenters should be cautioned that when running statistical analyses of concurrent TMS-fMRI data, the threshold for their analysis should be lowered and brain masking removed to determine if statistical irregularities resemble coil discharge artifacts at the site of the TMS coil or temporally targeted slices exist. If these artifacts are discovered in their statistical maps, the ICA results can be used to remove additional components that might capture these sources of signal artifacts.

### 3.4 Activation of frontostriatal loops from TMS was hierarchical

**FIGURE 4:**
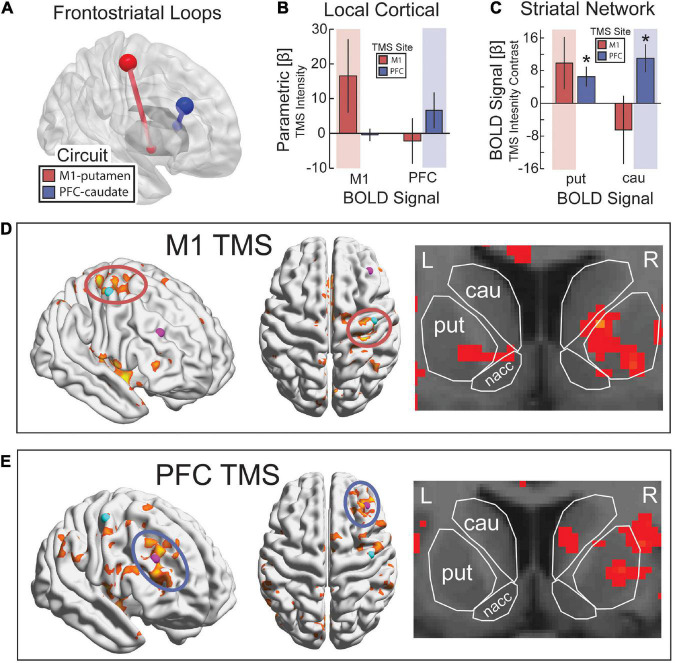
Targeting frontostriatal loops with continuous TMS-fMRI. **(A)** TMS to M1 (red) and PFC (blue) with anatomical projections to posterior putamen and dorsal caudate, respectively. **(B)** As TMS intensity was increased, we expected a parametric increase in evoked BOLD activity from TMS in the region of the cerebral cortex targeted by TMS (red background for M1 and blue background for PFC). **(C)** Parametric contrast of TMS intensity on evoked activity in putamen and caudate. Highlighted regions reflect the predicted increase in activity based on anatomical projections. Error bars are SEM. *p < 0.05. Statistical map for the parametric contrast of TMS intensity with TMS to M1 **(D)** and PFC **(E)**. *p* < 0.05, k > 500. **(D)** k > 500, **(E)** k > 20.

**FIGURE 5:**
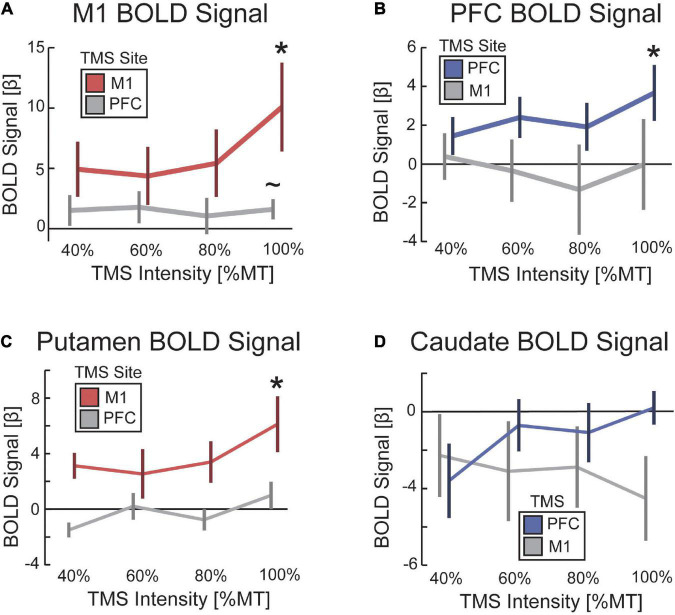
TMS increased evoked BOLD activity as a function of TMS intensity. The impact of TMS on the BOLD signal as a function of TMS intensity for **(A)** M1, **(B)** PFC, **(C)** putamen, and **(D)** caudate. Color-coding reflects conditions hypothesized to demonstrate a parametric increase in BOLD signal with TMS intensity. Red denotes TMS to M1 when reporting the BOLD signal in M1 and putamen. Blue denotes TMS to PFC when reporting BOLD signal in PFC and caudate. Error bars are standard error to the mean. *p < 0.05. ∼*p* < 0.1.

An experiment was conducted to validate whether our concurrent TMS-fMRI procedure was sufficient to capture activation under the site of TMS and in anatomically connected frontostriatal circuitry (Figure 4A). First, we hypothesized that TMS to M1 would evoke a BOLD response in M1 and TMS to PFC would evoke a BOLD response in PFC. To test this hypothesis, we ran a two-way ANOVA with a between-participant factor of the site of TMS and a within-participant factor of BOLD signal in the targeted and non-targeted regions. When analyzing the parametric effect of TMS intensity on the BOLD signal, we found a small interaction effect that was not significant [*F*_(1,13)_ = 2.70, *p* = 0.125, η^2^ = 0.21] (Figure 4B). However, we found a trend-level main effect of the site where the BOLD signal was estimated [*F*_(1,13)_ = 3.534, *p* = 0.083, η^2^ = 0.27]; M1 showed a large effect size for TMS to both M1 [*t*(7) = 2.74, *p* = 0.029, d = 0.97] and PFC [*t*(6) = 1.93, *p* = 0.10, *d* = 0.73], although this effect was only significant for M1 and was trend-level for PFC (Figures 5A, B). By comparison, *post hoc t*-tests revealed that PFC was only activated by TMS to PFC [*t*(6) = 2.55, *p* = 0.043, *d* = 0.97], but not by TMS to M1 [*t*(7) = −0.0045, *p* = 0.99, *d* = 0.002]. We also hypothesized that TMS at 100% of the resting motor threshold would result in the largest effects on the BOLD signal in the stimulated region. The same two-way ANOVA but for only the highest intensity of TMS revealed a medium effect size that was significant [*F*_(1,13)_ = 6.67, *p* = 0.023, η^2^ = 0.51]. These findings support our hypothesis that TMS would drive a TMS-evoked BOLD signal in the targeted location. Furthermore, our findings suggest a cortical hierarchy in which PFC occupies a higher location (Verstynen et al., 2012; Choi et al., 2018). The defining characteristic of a hierarchy is an asymmetry in projections such that higher-order regions project to lower-order regions, but not vice versa (Badre and D’Esposito, 2009). Our results suggest a functional hierarchy in that TMS to PFC spread to more caudal regions (Figure 4E), whereas TMS to M1 did not spread rostral into PFC (Figure 4D).

Second, we hypothesized that TMS to M1 would evoke a BOLD signal response in the anatomically connected site in the putamen, whereas TMS to PFC would evoke a BOLD signal response in the dorsal head of the caudate nucleus. We constrained our analysis to these specific striatal targets based on previous work using parallel TMS-PET (Strafella et al., 2001, 2003). To test for these remote effects, we ran a two-way ANOVA on the parametric increase of BOLD signal with TMS intensity using a between-participant factor of the site of TMS and a within-participant factor of the site in dorsal striatum for which BOLD signal was estimated. We found a significant interaction between the site of TMS and the site in the dorsal striatum where the BOLD signal was estimated [*F*_(1,13)_ = 5.92, *p* = 0.032, η^2^ = 0.49] (Figure 4C). *Post hoc t*-tests revealed that TMS to M1 showed a medium-sized effect on evoked BOLD signal in putamen that was not significant [*t*(7) = 1.55, *p* = 0.17, *d* = 0.55] and no effect on the BOLD signal in the caudate [*t*(7) = −0.78, *p* = 0.46, *d* = 0.28]. However, the parametric response was greater in putamen than caudate [*t*(7) = 2.20, *p* = 0.064, *d* = 0.78], and the highest amplitude of TMS output showed a significant BOLD signal response in the putamen [*t*(7) = 3.04, *p* = 0.019, *d* = 1.08] that was not present in the caudate [*t*(7) = −2.05, *p* = 0.08, *d* = 0.72]. Similarly, *post hoc t*-tests revealed that TMS to PFC showed a significant parametric increase in BOLD signal in both putamen [*t*(6) = 2.70, *p* = 0.036, *d* = 1.02] and caudate [*t*(6) = 3.20, *p* = 0.019, *d* = 1.21]. The parametric effect of TMS intensity on the BOLD signal followed the expected pattern in the putamen (i.e., a linear increase in BOLD with increasing TMS intensity), but the caudate showed a decrease in BOLD signal with low-intensity TMS and only a minimal increase with high-intensity TMS (Figures 5C, D). The statistical maps of the parametric effect of TMS to M1 (Figure 4D) and PFC (Figure 4E) revealed that TMS produced an increase in the BOLD signal under the coil as a function of TMS intensity. As hypothesized, TMS to M1 preferentially activated the posterior putamen, whereas TMS to PFC preferentially modulated the dorsal caudate nucleus. In support of hierarchical anatomy, TMS to M1 resulted in parametric modulation of the putamen, whereas TMS to PFC showed this effect in both caudate and putamen.

## 4 Discussion

The methodological framework established in this paper builds on previous efforts by providing the additional groundwork for implementing concurrent TMS-fMRI experiments and replicating this environment across labs. Our results demonstrate that, given the proper considerations, carefully-tailored TMS during continuous fMRI and thorough preprocessing can produce artifact-free data that reliably demonstrates activation in the targeted region and regions connected to it. Most TMS-induced artifacts are reduced through judicious timing of TMS during scanning and thorough attention to removing known artifacts in the preprocessing of the fMRI data. When TMS is delivered concurrently with continuous fMRI, we recommend that TMS is delivered during the crusher gradient prior to RF excitation. In our approach, contaminated slices were interpolated with the prior and subsequent slices in time. We used ICA to remove residual TMS coil discharge artifacts in the volumes acquired during TMS and residual signal dropout around the TMS coil. Artifacts around the location of the TMS coil could be reliably identified using a multiple regression model of instantaneous signal change. We caution researchers that when TMS is delivered concurrent with a standard EPI timing scheme, TMS artifacts in brain regions under the coil can persist for up to 8 s after TMS. We posit that these artifacts are due to leakage current as the capacitor recharges. A FIR model can be used to verify whether any discovered activation follows the canonical HRF or whether it tracks with an expected signal dropout radiating from the location of the TMS coil at the time of discharge. Our results also provide causal evidence that TMS delivered to PFC activates both the region under the coil and anatomically connected regions in the dorsal striatum in line with previous subcortical-targeted TMS-fMRI studies (Hermiller et al., 2020; Sydnor et al., 2022). Furthermore, we found causal evidence supporting the rostrocaudal hierarchy in PFC, characterized by asymmetrical projections toward more posterior sites (Verstynen et al., 2012). TMS to PFC spread to caudal regions in the cortex (M1) and striatum (putamen), whereas TMS to M1 did not spread to rostral regions in the cortex (PFC) or striatum (caudate).

### 4.1 Methodological recommendations

In our experiment, we chose to deliver TMS during the first crusher gradient prior to RF excitation of slice acquisition. However, if leakage current, eddy currents, or coil vibrations are significant sources of instability, then stimulating during a crusher gradient period may produce local artifacts around the TMS coil that last for several milliseconds, rendering the RF period susceptible to the artifacts. In this scenario, the optimal time for TMS shifts from during crusher gradients to during k-space readout because the loss of a single slice in the current volume is less costly than errors that can persist across the entire volume for multiple TRs. Furthermore, visual inspection of fMRI data may be misleading. Statistical analysis of the visually imperceptible TMS pulses during the crusher gradients revealed a systematic signal distortion. Thus, it might be preferable to target k-space read-out in slices acquired just after those within the range of the TMS coil artifact. In our dataset, this would roughly equate to slices 10–16. Furthermore, targeting the center of k-space provides systems without sub-millisecond precision to deliver continuous TMS-fMRI; for example, there are plus or minus 17 ms of allowable error when targeting the center of k-space in a 50-ms slice acquisition where the first 16 ms are devoted to crusher gradients and RF excitation.

For a typical (3 mm)^3^ spatial resolution, the k-space read-out takes approximately 75% of the total time required per echo planar image; the RF and crusher gradients account for the other 25%. In full k-space sampling (i.e., no partial Fourier), high spatial frequency information is collected first, then low frequency (peaking at the echo time, TE), and finally, high frequency again. Given that the majority of signal and contrast in fMRI is acquired during the low-frequency center of k-space, it is theoretically possible to align TMS to the end of the read-out period, omit these high-frequency k-space lines, and reconstruct a final image using the standard partial Fourier approach. This would recover a usable image, albeit slightly different from the image that would be acquired with a full k-space acquisition in the absence of TMS pulses. Alternatively, the corrupted portion of k-space could be linearly interpolated with k-space from neighboring slices or from volumes adjacent in time. It is also worth noting that targeting TMS early in the read-out period, immediately following the slice excitation pulse, maximizes the time between the TMS pulse and the RF pulses of the subsequent slice. Thus, if leakage/eddy currents and mechanical vibrations in the TMS coil are of concern and it is desired to sample EPI continuously, applying TMS as early as possible relative to the next pair of RF pulses will minimize later artifacts at the expense of a corrupted current slice.

### 4.2 Does TMS drive BOLD activity under the coil?

We found evidence that TMS can elicit activity in the region under the coil. These effects are shown to be independent of potential coil discharge artifacts for multiple reasons. First, the FIR model shows that the activation follows a canonical HRF curve and does not resemble the coil discharge artifact pattern observed prior to ICA. Second, the spread of activation followed anatomically plausible pathways. For example, TMS to PFC spread to M1, whereas the reverse was not true for M1.

Furthermore, the spread of activation with TMS to PFC spread posteriorly primarily within the middle frontal gyrus, and there was less spread within the inferior and superior frontal gyri. Third, previous studies of TMS have shown an increase in BOLD activation in bilateral auditory cortex (related to the audible click of the TMS coil) and bilateral insula [presumably related to the discomfort of TMS (Iannetti and Mouraux, 2010)]. Consistent with these previous findings, our parametric maps show that activity within the insula increased as a function of TMS intensity. However, we did not see increased activation in the auditory cortex, likely because the sound of the TMS click is not discernibly different between such similar intensity levels with ear plugs, cushions pressed against the ears to pack the head in place, and the noise of the MRI scanner. Investigation of striatal activation as a function of TMS intensity and site revealed that TMS to M1 spread to bilateral putamen, whereas TMS to PFC spread to ipsilateral caudate and putamen. These findings are consistent with the strong reciprocal connections between bilateral primary motor cortex, whereas hierarchical control from PFC to M1 is unilateral.

Experiments using concurrent TMS-fMRI have found conflicting evidence with regard to the local impact of TMS on the cerebral cortex. While TMS to M1 is generally accepted to increase the BOLD signal (Bohning et al., 1999, 2000, 2003; Bestmann et al., 2003b), the impact of TMS to the prefrontal cortex on the BOLD signal is less consistent (Rafiei and Rahnev, 2022). For example, a recent study did not find a univariate increase in BOLD activity under the TMS coil but instead found a multivariate pattern of activity that could classify the presence and frequency of TMS in the BOLD activity under the coil (Rafiei et al., 2021). We speculate that the persistence of TMS coil discharge artifacts might yet be underestimated. First, most statistical analyses *via* a GLM mask the brain and do not consider activation outside the skull. While this is a reasonable approach for fMRI studies without TMS, the presence of the TMS coil provides a unique situation where statistical modeling is useful. A region of interest around the TMS coil and above the scalp revealed a canonical pattern of signal dropout and recovery that lasted for many seconds, on the order of 6–8 s, for our particular experimental protocol. This same TMS coil artifact pattern was present within the cerebral cortex, except that it was superposed with the canonical HRF. By combining ICA with a multiple regression model to include a time-locked signal proximal to the TMS coil, we could more accurately estimate the sources of artifact and remove them from the data. Furthermore, continuous TMS during fMRI acquisition may have enabled us to more accurately characterize and remove the TMS coil artifact by collecting more data around the time of TMS.

In addition, previous studies have noted that the effect of TMS on BOLD activity in the targeted cortical region is more variable when stimulation intensity is beneath the participant’s resting motor threshold (Bestmann et al., 2003b; Hanakawa et al., 2009; Navarro de Lara et al., 2017). When TMS was targeted to the motor cortex, a linear increase in BOLD activation was observed as a function of stimulation intensity, however, the activation for subthreshold intensities did not reach statistical significance (Hanakawa et al., 2009; Navarro de Lara et al., 2017). These previous findings are consistent with the results of our experiment, wherein suprathreshold stimulation (100% resting motor threshold) of M1 and PFC revealed a significant increase in local BOLD activity that was not observed for subthreshold stimulation. In our data, we observed a qualitative deviation from linearity for TMS at 100% that was more pronounced in M1 than in PFC. One possible explanation for the variable effects of subthreshold TMS is the differential activation of excitatory and inhibitory neurons within the targeted region, as suggested by paired-pulse TMS experiments that found increased inhibition with low-intensity subthreshold TMS (Awiszus et al., 1999; Fisher et al., 2002). These effects likely vary across different cortical regions. Future studies that use invasive recording and cell-type classification are required to characterize better the neural response to subthreshold TMS across the brain.

### 4.3 Concurrent TMS-fMRI in cognitive neuroscience

All scientific methods have strengths and weaknesses. Although concurrent TMS-fMRI is technically challenging, the integration of causal and correlational methods provides a powerful tool to advance our understanding of the human brain. Incorporating both techniques in the same experimental study combines TMS’s precise spatial and temporal resolution with the strong spatial resolution and broad coverage afforded by fMRI. Each method complements the other and strengthens the conclusions we can draw from the results above and beyond using either method in isolation. Whereas behavioral studies that use TMS can provide valuable causal evidence about the consequences of stimulation on cognition (Pascual-Leone et al., 1992; Pitcher et al., 2007; Polanía et al., 2018; Riddle et al., 2019, 2020a; Bergmann and Hartwigsen, 2021), they cannot characterize the effect of stimulation on neural activity. Collecting concurrent fMRI data addresses this gap and can be used to investigate novel questions regarding the physiological effects of TMS, the relationship between TMS effects on brain and behavior, and the causal impact on functional brain networks that underlie cognitive function.

As discussed, concurrent TMS-fMRI can be used to interrogate local effects in the region under the TMS coil. Beyond these local effects, the approach is particularly well-suited to study neural dynamics in large-scale brain networks: researchers can causally perturb neural function in one area and observe the propagation of signals throughout the brain. Consistent with our experimental findings, previous work that applied TMS during the resting state has found that stimulation to one node in a network can elicit increased fMRI signal in anatomically remote regions in the same network (Bestmann et al., 2003b, 2004, 2008a; Denslow et al., 2005; Ruff et al., 2009; Bestmann and Feredoes, 2013; Hawco et al., 2018; Oathes et al., 2021; Sydnor et al., 2022). Although resting-state studies provide an important first step to characterize the local and distributed effects of TMS, combining concurrent TMS-fMRI with experimental tasks and behavioral measures is critical to studying cognitive function. A growing body of work has shown that the remote effects of TMS to one region can vary in a state-dependent manner (Ruff et al., 2006, 2008; Bestmann et al., 2008c; Blankenburg et al., 2008; Driver et al., 2009; Hawco et al., 2017; Hermiller et al., 2020). This provides a methodological advantage over parallel TMS-fMRI, wherein offline TMS [e.g., theta-burst stimulation (Lee and D’Esposito, 2012; Gratton et al., 2013; Cameron et al., 2015; Rahnev et al., 2016)] is performed before a participant enters the MRI scanner. The temporal resolution of concurrent TMS-fMRI allows stimulation to be applied during different task conditions or at different time points within a trial to probe how the remote effects change under different cognitive demands. Using the procedure described here, preliminary work from our lab has found that experimental timing can be aligned with the crusher gradients, and stimulation-related artifact components can be identified independently of task-related hemodynamic responses.

Concurrent TMS-fMRI, therefore, provides a “physiological probe” to obtain causal evidence for temporally-specific functional connections within brain networks that cannot be addressed with fMRI alone. While relatively few concurrent TMS-fMRI studies have moved beyond collecting data during the resting-state or outside the motor system, the findings from previous work highlight the inferential power of concurrent TMS-fMRI. For example, in one study TMS to the right intraparietal sulcus (at a site implicated in top-down visual attention) elicited remote fMRI activity in visual area V5/MT+, but only on trials where moving stimuli were present in the visual display (Ruff et al., 2008). In another study, TMS to the middle frontal gyrus during a visual working memory task elicited remote activity in posterior visual areas that were localized to category-specific regions (fusiform face area or parahippocampal place area) that matched the working memory content (faces or houses) (Feredoes et al., 2011). Critically, TMS to the middle frontal gyrus only influenced activity in posterior cortical regions when distractors were present, providing causal evidence for top-down frontal signals that modulate visual cortex to mitigate distractor interference (Feredoes et al., 2011). These findings provide fundamental new insights into top-down signals, frontal-striatal and cortico-thalamic circuits, and cortical hierarchies (Ruff et al., 2006, 2008; Badre and D’Esposito, 2009; Driver et al., 2009; Feredoes et al., 2011; Ruff, 2013; Heinen et al., 2014; Leitão et al., 2015; Badre and Nee, 2018; Bergmann et al., 2021; Gordon et al., 2022).

Consistent with theories that propose a rostral-caudal hierarchical gradient in the frontal cortex (Badre and D’Esposito, 2009; Badre and Nee, 2018), we found that TMS to PFC activated regions caudal to the stimulation site, but TMS to M1 did not activate regions rostral to M1. This hierarchical pattern of effects was also demonstrated in remote striatal activity such that TMS to PFC activated both the head of the caudate and the putamen, whereas TMS to M1 primarily activated the putamen (Verstynen et al., 2012; Choi et al., 2018). This is consistent with previous studies investigating how hierarchical cognitive control is anatomically instantiated in the hierarchical organization of PFC (Badre and D’Esposito, 2007) and corresponding frontal-striatal circuits (Verstynen et al., 2012; Choi et al., 2018). Task rules regarding higher-order contexts and control policies are represented in more rostral prefrontal cortex (e.g., anterior, middle frontal gyrus), while lower-order stimulus-response mappings are represented in more caudal frontal cortex (e.g., premotor and motor cortex) (Badre and D’Esposito, 2009; Badre and Nee, 2018). A hallmark of hierarchical processing is an asymmetrical influence among regions. Previous work has addressed this causal influence by studying patients with lesions (Badre et al., 2009) or by using off-line theta-burst TMS to create virtual lesions (Rahnev et al., 2016). In hierarchical decision-making tasks, frontal cortex lesions impaired decision-making at a level of representation dependent on the lesion location, as well as impaired decisions that required higher-order (more rostral), but not lower-order, levels of representations (Badre et al., 2009). This pattern implies a processing hierarchy in which hierarchical decision-making requires higher-order regions to exert influence over intact lower-order regions but not vice versa. Our TMS-fMRI results show a complementary asymmetry: online TMS resulted in activity that spread to lower-order (more caudal), but not higher-order, frontal cortex regions and the striatum (Figure 4). However, the influence of one region upon another likely does not follow a strictly unidimensional rostral-caudal gradient but may instead involve a more flexible hierarchical structure that depends on the nature of control demands (Nee and D’Esposito, 2016, 2017). Future studies that combine concurrent TMS-fMRI with hierarchical tasks will be able to characterize the precise causal relationship among regions in the frontal hierarchy, as well as the broader frontal-striatal network dynamics that support hierarchical control of behavior (Badre and Nee, 2018).

### 4.4 Limitations

One limitation of the present study is that TMS was delivered during the resting-state. While studies that deliver TMS in the resting-state can provide insight into trait-like properties of the targeted region or probe anatomical relationships between regions, the impact of TMS on cognition is context-dependent. Also, while most TMS studies have considered their findings in the context of the effects of TMS on either exciting or inhibiting neural activity, recent work demonstrates that electrical activity in the brain exhibits distinct neural oscillatory signatures (Riddle et al., 2020b), and targeting a single region with different frequencies of TMS results in distinct impacts on cognition (Thut et al., 2011; Hanslmayr et al., 2014; Riddle et al., 2019, 2020a).

In our experiment, TMS to PFC resulted in a parametric increase in BOLD signal as a function of TMS intensity. However, the mean change in BOLD signal across all stimulation intensities was shifted marginally negative, although not significantly negative. Because this pattern was not significant, we did not draw any definitive conclusion regarding this mean shift. As discussed, the delivery of TMS during the resting state does not consider context-dependent changes in the evoked BOLD signal.

Another limitation is that our experiments used single-shot EPI, whereas simultaneous multi-slice (SMS) EPI is becoming a widely used alternative for higher spatial-temporal resolution fMRI. The artifact patterns induced by TMS stimulation are predicted to have a greater spatial effect with SMS-EPI, almost certainly affecting all simultaneously acquired slices whenever a TMS pulse is delivered. A separate evaluation of concurrent TMS with SMS-EPI is warranted. We also note that SMS-EPI would not be practical with our hardware setup because we chose to use a single-channel birdcage coil for greater flexibility in TMS coil placement. While slices obtained with a very low SMS factor might be separated using the blipped CAIPI scheme alone, it is to be expected that a multi-channel receive coil with some heterogeneity along the slice direction would be a significant advantage for SMS-EPI. We have proposed and built a 50% scale prototype 6-channel “nested” birdcage coil that would offer the combined benefits of SMS-EPI and space for flexible TMS coil placement (Bradshaw et al., 2018).

Finally, the current study used a small sample size, and these findings should be interpreted as preliminary. Nonetheless, the statistical maps from our group analysis are promising in suggesting that these methods might prove useful to the field as a step toward improving data quality.

## Data availability statement

The raw data supporting the conclusions of this article will be made available by the authors, without undue reservation.

## Ethics statement

The studies involving human participants were reviewed and approved by University of California, Berkeley. The patients/participants provided their written informed consent to participate in this study.

## Author contributions

JR and MD: experimental design. JR and CM-F: data collection and analysis. DS: real-time hardware design and construction. DS, JR, and BI: artifact characterization. JR, BI, DS, and MD: wrote the manuscript. All authors contributed to the article and approved the submitted version.
