# Supplementary material for: A guide for concurrent TMS-fMRI to investigate functional brain networks
Source: Front Hum Neurosci. 2022 Dec 15;16:1050605. doi: 10.3389/fnhum.2022.1050605 (PMC9799237; doi:10.3389/fnhum.2022.1050605)
Supplement: Supplementary file 1 [file Data_Sheet_1.docx]

## Supplemental Material for Riddle J; Scimeca J; Pagnotta MF; Inglis B; Sheltraw D; Muse-Fisher C; D’Esposito M. “A guide for concurrent TMS-fMRI to investigate functional brain networks” *Frontiers in Human Neuroscience*, 2022.

## **Technical considerations for simultaneous TMS-fMRI**

Accurate MR imaging requires a homogeneous polarizing magnetic field, linear field gradients for spatial encoding, and carefully tailored oscillating magnetic fields with which to manipulate the magnetization. TMS involves the production of brief, intense magnetic fields over a subject’s scalp. When performed inside an MRI scanner there are several ways that the TMS can interact with the MRI hardware and pulse sequence. Applying a large, time varying magnetic field from a TMS coil can perturb one or all of the critical imaging magnetic fields. The potential for TMS to corrupt fMRI data was appreciated from the earliest concurrent TMS-fMRI studies [1-4], and significant efforts have been made to address the complex interaction of TMS and fMRI [5-7]. Most obviously, the TMS pulses of intense magnetic field are experienced by the nuclear spins and can perturb the MR image formation. The precise consequences of the interaction depend on such factors as the magnitude and spatial heterogeneity of the TMS magnetic field and the state of the nuclear spins at the time of the TMS magnetic field. Phase coherence is used to encode spatial information, which is different than spins at thermal equilibrium. Other artifacts can arise even when the TMS coil is not being pulsed. For example, the TMS coil, constructed of copper wire, has a magnetic susceptibility that is different to foam pillows or other items that may be placed around the head for comfort or support. Finally, there may be practical limitations in the TMS control electronics that cause unintentional, weak magnetic fields from the TMS coil even when the TMS coil is not supposed to be delivering magnetic fields.

In **Table 2** of the primary manuscript, we listed the artifacts and technical issues that can occur in fMRI data as a result of including a TMS coil in the setup, and/or conducting pulses of TMS during the fMRI acquisition. The artifacts have been categorized by their physical origins. Likely severity and order of importance are difficult to estimate because they are strongly influenced by the particular hardware choices as well as how the experiment is set up. We have given approximate temporal scales for each phenomenon in **Table 2** of the primary manuscript but wish to emphasize that the actual temporal nature can also depend on hardware choices, on the way the TMS coil is positioned relative to the subject’s head or in proximity to the MRI hardware, and so on. As we shall explain, some of these artifact sources can be mitigated through judicious timing. Some, however, may require reengineering of some aspects of either the TMS or the MRI hardware.

### **MRI artifacts caused by presence of a TMS coil**

*Magnetic susceptibility*

Copper is diamagnetic with a volume magnetic susceptibility only 7% greater than water. However, the presence of the TMS coil can produce a region of signal dephasing similar to the effects of venous sinuses and skull structures, and leads to slightly reduced T_2_* under the coil. With appropriate choices of plastic housing and potting compounds, a modern commercial TMS coil designed for use in an MRI scanner has a small effect on the main magnetic field homogeneity. Even so, motion of the head relative to the coil, especially when the coil is supported independently, may lead to increased signal variance in an EPI time series.

Magnetic susceptibility gradients arising from the TMS coil can be evaluated using phase-sensitive images, e.g., a multi-TE gradient echo scan with both magnitude and phase images collected. Even after shimming, the presence of a TMS coil will produce some reduction of T_2_* in the phantom or brain underneath. Comparing scans with and without the TMS coil on a phantom should show the evolution of T_2_* arising from the presence of the TMS coil. The static effects are generally weak for modern TMS coils. Phase images at multiple TEs will show the effects of magnetic susceptibility with better specificity than magnitude images, because magnitude images show the simultaneous effect of RF shading. Note also that scanning heats the imaging gradients, so shimming before each measurement is important to reset global phase effects.

*RF shading*

The copper windings tend to shield the transmission B_1_^+^ field, generally causing a low intensity region underneath the coil because the effective flip angle is reduced from its nominal value. There is awareness of the problem and new RF coils are being designed for use with TMS coils with B_1_^+^ shading in mind [8, 9]. Scanners with parallel transmit capability may allow a degree of RF shimming to compensate for the largest effects of RF shading but for fMRI, at least, the additional small variation in excitation flip angle under the TMS coil should be a minor effect. Temporal instabilities are usually a far greater concern.

Evaluation of RF shading can be performed with two gradient echo (GRE) data sets acquired at different excitation flip angles, to permit estimation of a B_1_+ map. Alternatively, dedicated B_1_+ mapping sequences are available on some platforms. While the effects of RF shading are expected to be relatively minor for conventional gradient echo EPI, there may be additional consequences for simultaneous multi-slice (SMS) EPI used for fMRI, and for diffusion MRI using spin echo (SE) EPI, both of which require more complicated and/or larger RF flip angles. In these cases, it is important to conduct dedicated testing on a phantom using the specific sequence(s) to be deployed in the presence of a TMS coil.

*Vibration*

The TMS pulse is applied as a brief, intense current through the TMS coil circuit. The generation of a magnetic field from the electric current through the windings produces a concomitant Lorentz force on the windings. This force can set into motion any of the possible vibrational and ballistic modes of the coil’s mechanical structure, one consequence of which is the audible “click” with each pulse. A typical figure-8 geometry coil is designed to achieve zero net force on its center of mass as well as zero net torque about its center of mass. In addition, the windings of the coil are potted in a rigid medium to reduce vibrations of the coil windings. The degree to which the TMS pulse is effective in driving these vibrations depends upon the natural frequencies of the vibrational modes, the frequencies of the TMS pulse which drives the vibrations and the damping constants associated with the vibrations. The closer the driving frequencies are to the mechanical vibrational mode frequencies the greater the vibration amplitude of the corresponding mode. Imperfections in the coil geometry and the potting of the windings therefore gives rise to small ballistic and vibrational motions.

The most obvious effect of coil vibration is the clicking sound produced by each TMS pulse. There is a slight change in the acoustic signature of a TMS pulse applied inside an MRI scanner compared to its sound outside the scanner, in the earth’s magnetic field, because of the additional Lorentz forces induced by the MRI magnetic field coupled to the TMS coil’s intrinsic field.

Mechanical vibrations of the coil may also cause image artifacts. Motion of the TMS coil, which has a slightly different magnetic susceptibility than both the surrounding air and the subject’s head, distorts the static magnetic field across the brain, thereby deteriorating image quality. Once again, the time course of mechanical vibrations, which depend upon the damping constants of the vibrational modes, will evolve in the period immediately following the TMS pulse. Characterizing the artifacts from mechanical vibrations is likely to be quite difficult for well damped, rapidly decaying vibrations. More problematic might be recoil of the phantom or subject’s head from the coil motion, or the subject’s unintentional reaction and movement to the sound pressure wave on the skull.

*Thermal drift*

Heating of the TMS coil with use has the potential to modulate all three of the aforementioned effects (magnetic susceptibility, RF shielding, and the vibration spectrum) primarily via a changing electrical resistance in the copper windings. Furthermore, the magnetic field generated by the coil may change as the resistance changes, if such changes are not taken into account in the power source. However, the resistance could be measured and the power source output modified as necessary to maintain a constant output. Changing resistance, arising out of Insufficient cooling, might thereby lead to a number of different low frequency modulations of signal underneath the TMS coil, and perhaps to modulation of the neural coupling as well (in the case of changing TMS output).

Temperature stability can be measured directly, e.g., using MRI-compatible thermocouples, laser thermometry and the like. Serial MRI scanning may also be able to report thermal drifts via phase-sensitive or magnitude EPI or short duration GRE or SE scans. Monitoring the temperature of return cooling air or water can also be a useful way to estimate overall coil heating characteristics, but this method is liable to miss any hot spots within the TMS coil. Measuring the TMS coil (or coil circuit) resistance should be able to detect hot spots as well as overall heating characteristics, but for most commercial TMS systems one is presently limited to the information reported at the TMS power supply.

*RF noise introduced via the TMS cable*

The TMS cable should be filtered effectively at the penetration panel of the scanner’s Faraday shield. Insufficient filtering will cause the injection of broadband white noise and the injection of discrete frequencies (electromagnetic interference, EMI) from outside the Faraday shield. Attention should also be paid to potential RF sources inside the magnet room, in case an otherwise benign low EMI source can be picked up by the TMS cable and carried into the magnet bore. Checks using an MRI vendor’s quality control routines for RF noise should suffice to measure the overall level of EMI with the TMS coil in situ, connected to its power source.

We use a vendor-supplied service routine, “RF Noise Check” to assess the degree of RF noise introduced into the scanner room via the filtered TMS cable. The test routine involves detecting a series of twenty 20 kHz bandwidths with carrier frequencies in overlapping 10 kHz carrier frequency steps from -100 kHz to +100 kHz relative to the central scanner on-resonance frequency of ~123 MHz. Any RF coil can be used for the test but it makes the most sense to use the same coil as used in TMS-fMRI experiments; in our case, a receive-only quadrature birdcage coil. We use a vendor-supplied bottle phantom for tests not involving a human subject or imaging. For imaging, a gel phantom is preferred to reduce the potential for standing waves in the medium. Four configurations were tested: 1. A phantom without TMS hardware, bed fully retracted from the magnet, 2. A phantom without TMS hardware, bed inserted in the magnet, 3. With TMS hardware on a phantom, bed inserted, and 4. With TMS hardware and a human subject, bed inserted. The initial test with bed retracted establishes a clean RF environment baseline without the waveguide effects of the magnet cryostat. The second test then establishes the baseline appropriate for scanning. Both are important because failing the RF Noise Check at step 1 makes it highly likely that the introduction of the TMS cable will exacerbate a preexisting problem. The third configuration tests the TMS cable and filtering. The entire TMS unit should be powered on for this test. If you are using peripheral equipment for the fMRI experiment then it is a good idea to enable these devices for this test at the same time. If the RF Noise Check fails, remove components systematically until the source of EMI is determined. The fourth step adds the extra antenna effect of a conductive human body. Again, the TMS unit should be powered on and all peripheral devices positioned as for a real fMRI experiment.

*RF spikes from friction inside the TMS coil*

As previously noted, pulsing the TMS coil creates a large Lorentz force on the coil windings, producing a characteristic click as the windings are torqued in both the magnetic field of the TMS coil itself as well as the scanner’s polarizing field, B_0_. Immediately after the TMS pulse, the windings will relax back to their equilibrium positions. Friction between the windings may produce low level EMI. There may also be weak electric fields that discharge with the small movements. In either case, weak RF interference would reduce thermal SNR and, if sufficiently severe and long lasting, may cause striping in EPI acquired in the period after the TMS pulse.

We conducted a test for a TMS pulse set to occur just after the three Nyquist ghost correction echoes and before the start of k-space readout. See later for details of TMS relative to pulse sequence events. The acquisition protocol consisted of fifty TR periods with no TMS pulse, during which the phantom was withdrawn from the birdcage coil, the magnet room lights switched off and the door closed. TMS pulses were applied at one slice, once per TR for volumes 51-150. Then a further 100 volumes were acquired with no TMS pulses. ROI plots clearly showed the effect of the TMS pulses in the target slice (**Supplemental Figure 1**, red box) but also show that later slices (yellow box) show higher numbers of EMI spikes than either a slice acquired before TMS in the current TR period (turquoise box) or very late in the TR period (green box). Eliminating the TMS pulses during the final 100 volumes returns the RF noise to baseline for all ROIs.


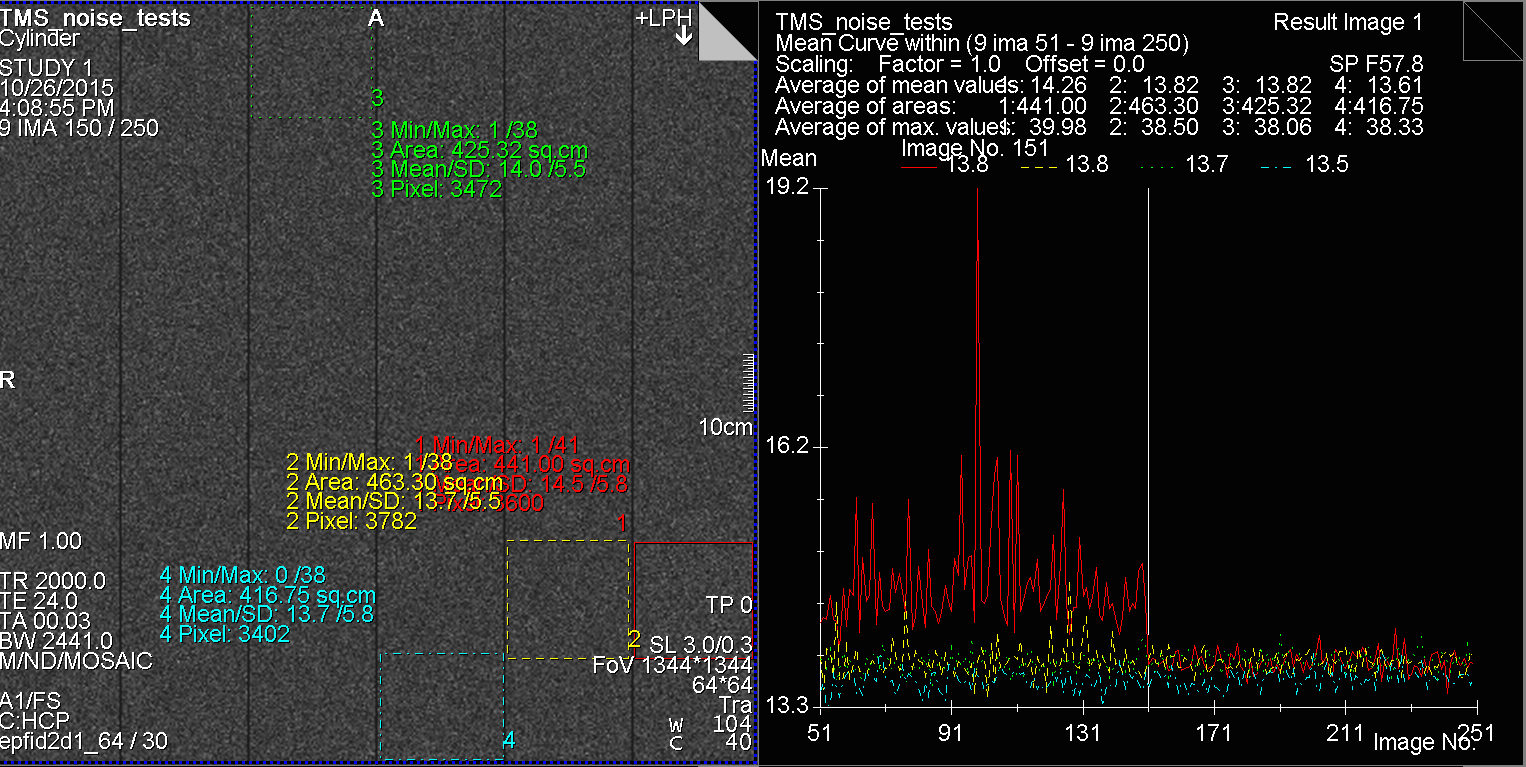


**Supplemental Figure 1:** Test for EMI arising from electrostatic discharges from the TMS coil following a pulse. The time series commenced with a phantom in the RF coil, and the phantom was removed during the first fifty volumes (TR = 2000 ms). When used, TMS pulses were timed to coincide with the slice position corresponding to the red box ROI in the left panel. TMS pulses were applied one per TR for volumes 51-150, then the TMS pulses were discontinued for volumes 151-251. Clear EMI arose immediately following the TMS pulses (right panel, red trace) and a few smaller peaks were detected in the following slice (right panel, yellow trace).

### **MRI artifacts arising in the TMS power supply**

*Leakage current*

Commercial TMS devices use large capacitors to produce the high current and rapid pulse rates required for TMS protocols. The capacitors must recharge after each TMS pulse, during which time the switch used to deliver a pulse is in its open state. Unfortunately, significant current can leak across the switch while in the open state, causing the TMS coil to produce a weak magnetic field during the inter-pulse delay [7].For our TMS system, a MagVenture system upgrade was installed which reduces the leakage current by using a relay-diode combination inserted in the TMS circuit that shorts the leakage current, reducing the leakage current by an order of magnitude (from 10 μA to 1 μA). While the intensity of leakage current magnetic fields is several orders of magnitude smaller than the TMS pulses and is of minor consequence from the perspective of brain stimulation, the spurious magnetic field caused by the leakage current is sufficiently large to perturb magnetization during EPI. Given the subtle changes in the BOLD signal often measured by fMRI experiments, these leakage currents can produce significant local image disturbances near the TMS coil, particularly in the 10 to 20 ms after a TMS pulse as the capacitor recharges. We take this delay into account in the specific timing used in our experiments.

*Asymmetric biphasic pulse*

Many TMS protocols now use biphasic pulses, for both neuroscientific (excitation properties) and engineering reasons. With a biphasic pulse shape, current that flows through the TMS coil is returned as capacitor voltage, causing partial recharging of the capacitors and decreasing the time delay before the TMS unit can deliver the next pulse. A further benefit is reduced heating of the TMS coil compared to a monophasic drive waveform, for a one-to-one comparison. (The shorter inter-pulse delay tends to mean that coil heating is still an issue for many common high frequency paradigms, such as theta burst TMS.) The biphasic pulse from a capacitor is not ideal, however. While the shape is approximately one full period of a sine wave, the second lobe has a slightly smaller amplitude than the first lobe. In conventional TMS-fMRI experiments, where the TMS pulses are timed to coincide with either dead periods or crusher gradients in the MRI pulse sequence, the asymmetry has minimal consequences. (Eddy currents in MRI hardware are considered below.) If the TMS pulse occurs at a time when there is transverse magnetization in the EPI sequence, an asymmetric TMS pulse will impart a net phase shift that varies spatially depending on the magnetic field produced by the TMS coil. This can be favorable, if the TMS pulse is coincident with crusher gradients, or undesirable, e.g., if the TMS pulse occurs during the k-space readout period. If one wishes to preserve the phase of the magnetization after a TMS pulse then a perfectly symmetric TMS pulse would, in principle, allow recovery and use of MRI signal in the k-space sampling period immediately after the TMS pulse, with just the line(s) of k-space coincident with the TMS pulse being irretrievably corrupted. Until larger sources of error are eliminated, however, it seems unlikely that such high precision concurrent TMS-fMRI would be practical, making the slight asymmetry in the biphasic waveform an issue that can be ignored for now.

### **MRI artifacts arising from coupling of TMS to the MRI hardware**

*Eddy currents in the MRI hardware*

The TMS coil is designed such that high current passing through its figure-8 windings generates a powerful magnetic field (around 1.5 tesla near the surface of the coil). Rapid switching of the current generates the desired rapidly oscillating electric field in the brain. Simultaneously, however, all conductive materials in the vicinity of the TMS magnetic field are prone to induced electric fields that flow in circular patterns. These eddy currents are likely to be problematic in any MRI hardware containing large metal components, with the magnet’s steel cryostat the largest component of concern. Copper strips in RF coils, RF shields, patient bed components and other items cannot be discounted *a priori*. The magnitude of any eddy currents will depend on the distance of the TMS coil to the conductive component, while the time constants of any eddy currents thus produced will depend on the geometry and electrical conductivity of the structure.

Eddy currents arising from the pulsed magnetic field gradients used for spatial encoding in MRI are reduced through gradient coil design (i.e., active shielding) and with pre-emphasis drive waveforms applied to the gradient amplifiers. While there are no similar controls for the TMS-induced eddy currents, it is worth noting that biphasic (approximately sinusoidal) TMS pulses tend to balance the induced fields in conductive structures, akin to crude pre-emphasis. Indeed, a biphasic diffusion-weighted gradient pattern is often used intentionally to minimize eddy current effects in diffusion-weighted imaging [10]. A fully symmetric TMS pulse shape is likely to self-compensate to a greater degree than an asymmetric pulse. In either case, however, eddy currents can be minimized by ensuring that the TMS coil is as far from the magnet cryostat as possible, and by ensuring that equipment containing metal is kept to a minimum, e.g., use plastic components to support the TMS coil.

*Receive coil to TMS coil inductive coupling*

Most modern MRI scanners use receive-only RF coils for head imaging. These coils are tuned to the Larmor frequency for protons and matched to a load representative of a typical human head. Inserting other materials into the coil, especially electrically conductive materials, can change the tuning and loading of the coil and degrade its performance. The TMS coil may generate a capacitance that alters the MR receiver coil’s tune and match. The global performance of the RF coil would then degrade. Imaging methods that are close to the thermal noise limit, such as diffusion-weighted imaging and highly accelerated anatomical scans, would be most affected by the degradation in receive sensitivity. The SNR of individual echo planar images would also suffer, but since fMRI experiments are generally assumed to be in a regime limited by physiologic signal fluctuations or motion effects, and given the constant nature of the degradation over an EPI time series, the consequences of lost sensitivity may not be severe for fMRI.

*Ring-down in receive coil decoupling electronics*

As noted in the section on eddy currents, a changing magnetic field creates a changing electric field in electrical conductors. Thus, when the TMS coil is pulsed, large electric fields may be generated inside any conductive loop, including electrical circuits. The receive-only RF coils are usually closed circuit, that is, they are capable of receiving a signal, and are only temporarily gated out (open circuit) when the transmission B_1_ field is pulsed. On most modern scanners the receive decoupling is achieved via dedicated gating signals sent from the scanner pulse sequence controller to dedicated decoupling circuits on the receiver coil. Hence, unless the receive coil is also gated during pulses of TMS, the TMS pulses may generate currents in the RF coil loops that cause electronic components to oscillate or even to fail completely. The fact that the TMS pulses are not at the tuned frequency of the RF coil may not matter if the currents or voltages induced are large. In the case of component ringing, we can expect the RF coil performance to be degraded until the electric fields induced by the TMS pulse have fully dissipated.

We have not performed a systematic investigation of ring-down in RF coils, in part because we are reluctant to risk breaking our RF coils. Ideally, one would use custom test coils that can be repaired easily and cheaply. In one test using a biphasic 250 microsecond TMS pulse timed to coincide with the center of k-space, we observed a very strong striping pattern that may have been caused by ring-down in the coil (**Supplemental Figure 2**). It is important to note, however, that EMI spikes from coil friction (see above) could also produce such an effect. That said, we did have one unexplained RF coil failure which required a repair by the manufacturer, and so we caution experimenters to be mindful of the TMS coil position relative to the receive coil electronics when designing an apparatus and protocol.


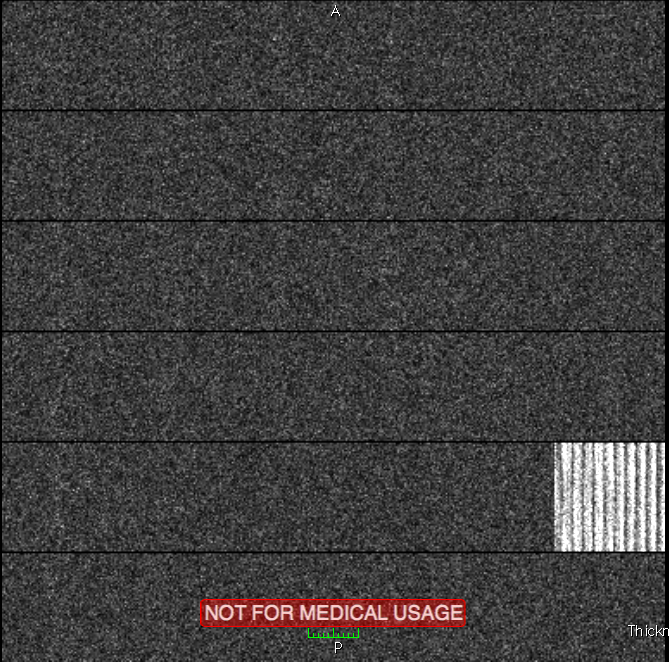


**Supplemental Figure 2:** Test of ring-down in a custom receive-only birdcage coil. A figure-8 TMS coil was suspended from the upper rungs of the birdcage coil several cm above a cylindrical phantom. The EPI acquisition was initiated and then the phantom was pushed out of the birdcage coil so that only noise images would result for the remainder of the time series. Biphasic TMS pulses of duration 250 microsec at 70% maximum stimulator output were applied, one TMS pulse per TR. Pulsing the TMS coincident with the k-space acquisition produced bright banding in the corresponding slice, as shown here. Subtle EMI stripes can be detected in other slices, suggesting either long-lived electrostatic discharges in the TMS coil or some long latency ring-down effects in the RF coil electronics.

*Transmitter coil to TMS coil inductive coupling*

Whole body transmitter coils must work efficiently over a wide range of subject body sizes. The mass and dimensions of the subject determine the dielectric load experienced by the transmitter coil. Introducing a TMS coil and its cable is likely to change the loading characteristics, as for the receiver coil considered above, and may lead to a slight performance degradation of transmission B_1_ field. Generally, however, the transmit coil voltage is calibrated at the start of each imaging session so that the variable subject load is compensated to a first approximation.

*Ring-down in transmit coil decoupling electronics*

Pulses of TMS magnetic field have the potential to create significant current in electrical circuits of transmit RF coil electronics as well as receive coil electronics. Unlike receive coils, however, the transmit coil is generally open circuit except when the scanner is instructed to pulse the transmission field. Furthermore, for body transmit coils as used on most MRI scanners, the size of the transmit coil and the greater distance of the TMS coil from the transmission coil components suggests that any ring-down in the transmit coil will be far less than that in the receive coil. However, experimenters using local or head-only transmit-receive RF coils should be mindful of the potential for unwanted coupling and aberrant behavior.

### **MRI artifacts arising from coupling of TMS to the EPI pulse sequence**

*Perturbation of M_0_ via an effective precessional field*

The application of a TMS pulse of ~1.5 T at the TMS coil surface, combined with a polarizing magnetic field of ~3 T, produces a large effective field experienced by protons in the vicinity of the TMS coil that is inclined at an angle to the MRI magnet axis. (The angle of the effective field depends on the inclination of the TMS coil to the MRI magnet axis.) Each TMS pulse lasts 250-300 microsec, compared to a Larmor frequency at 3 T of more than 120 MHz. The TMS pulse duration is thus more than 30,000 times greater than the precessional period of the spins under B_0_. Whether this makes precession about the effective field an important factor depends on the specifics of the MRI pulse sequence and on the TMS pulse shape. In the case of biphasic TMS pulses, the relatively long pulse duration suggests that the magnetization should behave adiabatically and simply follow the effective magnetic field. If entirely adiabatic, the magnetization, once exposed to the TMS pulse, should return to its initial state as the magnetic field associated with the TMS pulse returns smoothly to zero. This return to the prior state depends, however, upon the assumption that any irreversible effects (T₁ and T₂ decay) are negligible during the application of the TMS. Although the time scales associated with the irreversible effects are large compared with the length of the typical TMS pulse (T₂ values for brain tissues are approximately three decades greater than the duration of the TMS pulse and T₁ values are at least four decades greater), there may be small residual irreversible effects that are large enough to compete with the BOLD signal. We also note that there is a slight asymmetry in a biphasic TMS pulse produced from a capacitive discharge, so that a perfect identity transformation cannot occur. Monophasic pulses need special consideration for their effects on M₀.

While we have not yet run tests of M_0_ perturbation, we have considered some experiments that might determine whether precession about an effective field is an issue for any given TMS pulse shape and intensity. We suggest running a train of 5-10 TMS pulses (or bursts) at the experimentally desired rate (e.g., 4 pulses per sec, repeated every second) then sampling the steady-state magnetization using single-shot EPI with a 90-degree excitation flip angle; one single image volume acquisition, or even a single slice perpendicular to the TMS coil. The image SNR should be compared to EPI acquired without prior TMS, at thermal equilibrium. The EPI acquisition should be timed to avoid vibrations and other instabilities from the TMS pulses, but the post-TMS delay should be short compared to the sample T_1_ so that, in the case of perturbation, the longitudinal recovery towards M_0_ is small. And, as with most imaging-based tests, it is important to use a phantom that minimize the possibility of standing waves. Even some gel phantoms may support oscillations that persist for tens or hundreds of milliseconds. It is probably best to ensure an air gap between the TMS coil and the phantom. Simultaneous vibration measurements of the phantom may also be required to clearly establish the mechanism of signal reduction, if one is observed.

*TMS during RF pulses*

Applying TMS pulses simultaneous with RF pulses in the EPI sequence will cause artifacts in the current slice, is likely to cause artifacts in slices adjacent in time and space, and will generally perturb the T_1_ steady state into later TR periods. The magnitude of both the current slice artifacts, the effects on subsequent slices, and the persistence of residual effects to later TR periods will depend on the intensity of the B₁ field during the TMS pulse, the timing of the TMS relative to EPI slice ordering, and the range of T₁ values in the subject’s head. The entire situation will be further complicated whenever significant head motion is coincident with the TMS and RF pulses. Thus, we suggest that pulsing the TMS during RF pulses is avoided as a general rule. Care must also be used when TMS pulses are timed to occur just prior to RF pulses, in case any of the effects considered above, such as leakage currents, persist and perturb the B₁ field.

We provide a demonstration of the effect of TMS pulses applied during RF pulses in **Supplemental Figure 3**. TMS pulses were incremented through the entire pulse sequence for EPI, one TMS pulse per TR. The effects on a slice directly under the TMS coil are depicted in the yellow trace on the right-hand panel of the figure. TMS applied during the Gaussian fat saturation pulse traces a Gaussian shape in the TR-by-TR perturbation. The slice select RF pulse is sinc-shaped and broadcast in concert with a linear gradient. Hence, TMS during the slice select RF pulse traces the square-shaped frequency profile of the RF pulse and linear gradient pair.


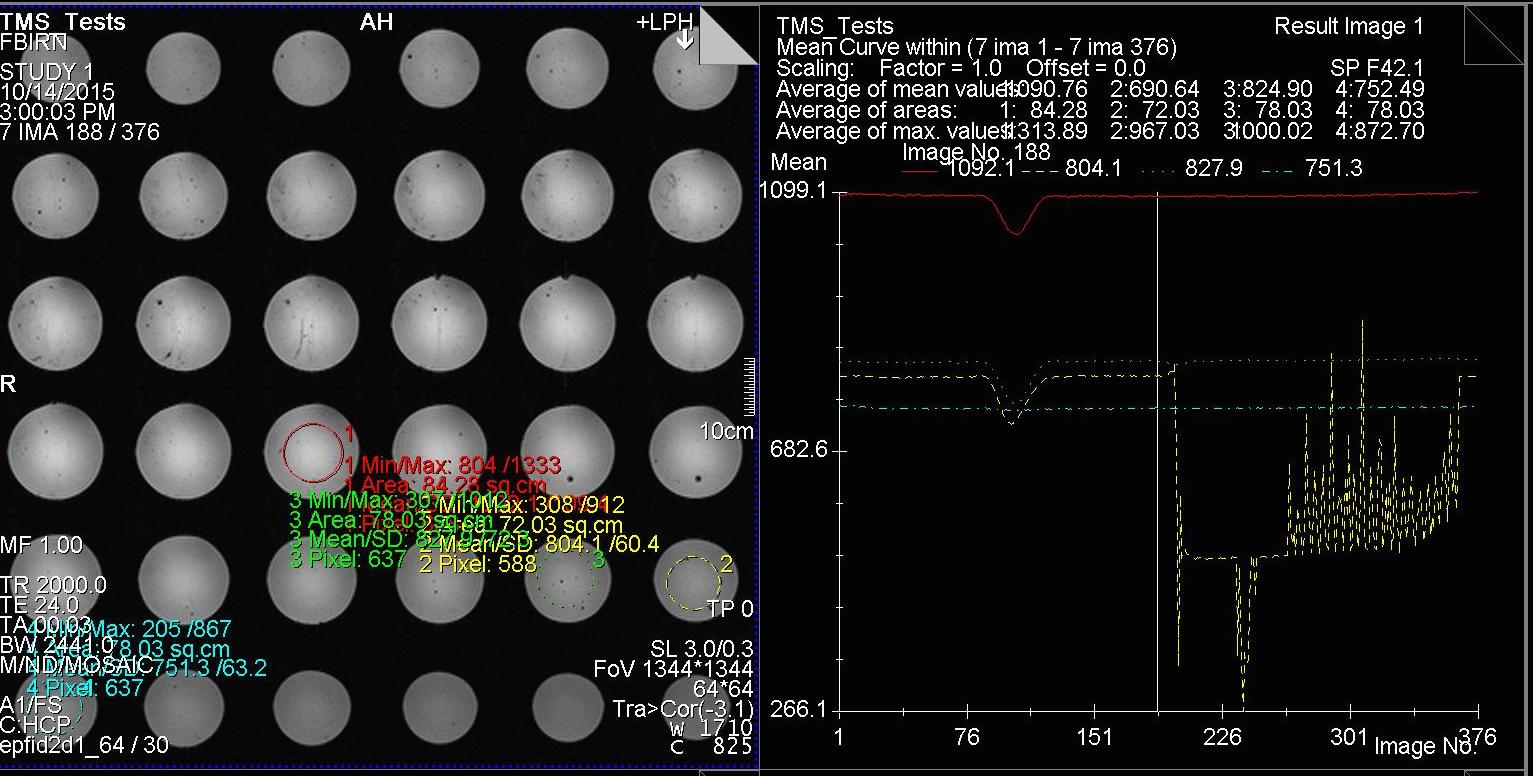


**Supplemental Figure 3:** The effects of bipasic TMS pulses of duration 250 microsec applied during a standard single-shot multi-slice EPI sequence. A single TMS pulse per TR = 2000 ms at 70% maximum stimulator output was applied for 376 increments of 0.1 ms relative to a reference time defined towards the end of the preceding slice, i.e. 376 repetitions of the full multi-slice EPI sequence. The absolute timing information is not critical. This example shows how a single TMS pulse has different effects depending on its timing relative to the EPI pulse sequence events. **Left panel:** The TMS coil was positioned on the top-front of a spherical FBIRN gel phantom, approximately at the position of the bottom three rows of slices. A gel phantom was preferred to a water phantom to reduce the possibility of long-lived standing waves from gradient vibrations. The slices were acquired in ascending order, i.e. from the bottom-right corner to top-left. Four ROIs are selected. **Right panel:** The TMS pulses were timed to coincide with the slice sampled with ROI2 (yellow dashed trace). The TMS pulse interacts only with the slice selection and k-space readout period of this slice. However, when the TMS pulse coincides with the Gaussian fat saturation pulse it also affects later slices (green trace from ROI3 and red trace from ROI1) but not the slice immediately prior to it (turquoise trace from ROI4). Note, however, that a TR much shorter than 2000 ms might have caused perturbation in ROI4 for subsequent volumes.

*Corrupted k-space*

The TMS magnetic field causes a phase to be imparted to transverse magnetization. The spatial heterogeneity of the TMS field as well as its far greater intensity, compared to a linear field gradient as used for MRI spatial encoding, will produce scrambled phase across most of the subject’s head. If a biphasic TMS pulse were perfectly symmetric we might expect it to act like a (very intense) gradient echo that leaves no net phase shift at the termination of the pulse. We might then be able to reject as little as a single line of k-space (a single frequency encoding period), given a TMS pulse of < 300 microsec and typical EPI echo spacing of around 500 microsec. (Ramp sampling periods would also need to be avoided.) The corrupted k-space line might then be replaced with the average of the two adjacent lines, or using some other strategy such as with parallel imaging. Given the asymmetry of TMS pulses produced by capacitive discharge, however, there will always be a non-zero residual phase imparted at the end of the TMS pulse, and this phase shift will corrupt the current and all subsequent k-space collection in the current slice. If the TMS pulse is timed to occur in the latter portion of the acquisition window, it may still be possible to recover the current slice using the data reconstruction methods used in partial Fourier imaging. The earlier in the acquisition period the TMS pulse occurs, the larger the corruption is likely to be. It is particularly important to avoid pulsing during the high signal, low frequency central portion of k-space or the entire slice will be corrupted, and partial Fourier reconstruction approaches will not work.

Whenever a slice is perturbed, it is important to consider any concomitant effects. These are most easily understood in the case of simultaneous multi-slice (SMS) EPI, where the cost of pulsing TMS during MRI sampling is then R slices, for an SMS acceleration factor R. Whether SMS or conventional multi-slice EPI, however, we must also assess the effect of the current, corrupted slice on successive acquisitions through slice crosstalk, i.e., through the well-known partial overlap of trapezoidal slice profiles. In general, contiguous slice ordering has been shown to exhibit fewer deleterious motion effects (e.g., banding) than interleaved EPI slices. In the case of simultaneous TMS with EPI, however, it may be that the artifacts arising from crosstalk negate the head motion benefits. Each situation must be considered separately. We might employ tactics such as targeting TMS pulses to occur when slices of low interest are being acquired, e.g., the most inferior or most superior slices, for a whole head axial prescription.

The effects of TMS pulses during the initial portion of k-space readout can be seen in Supplemental Figure 3. The k-space readout period begins at around image number 250 in the right panel. The final image, number 376, occurs 12.6 ms after the start of k-space sampling at image 250. For an increment of 0.1 ms and TE of 24 ms, the k-space center would have occurred at around image number 460 had the series been continued. Note how the image artifact quantified by the yellow ROI is punctate for the k-space edges (high spatial frequencies) but starts to curve as the TMS pulses move towards the high SNR, low frequency center of k-space.

*Flow/diffusion weighting*

In the previous section we considered only the direct effects of the TMS magnetic field on k-space, assuming static magnetization. The assumption of static magnetization may hold for TMS pulses of < 300 microsec, but given the amplitude and heterogeneity of the TMS magnetic fields we should not ignore the possibility of significant additional phase imparted to any spins moving through the TMS magnetic field. Diffusion weighting effects may be important for spin echo BOLD imaging, for example, if TMS pulses are timed to occur in only one-half echo time period, or for gradient echo EPI if the TMS is targeted to occur in any “dead time” between the slice selection and the start of k-space acquisition.

### **Summary**

The artifacts produced by leakage currents from the TMS power supply, eddy currents in MRI scanner components and vibrations of the TMS coil in response to a pulse are more or less indistinguishable from each other in their effects upon the MR images. Each will generate signal dropout surrounding the 3D location of the TMS coil. The spatially localized dropout returns to baseline further from the source of the artifact. The time constants can also be expected to overlap. Pilot testing on phantoms leads us to conclude that the leakage current and/or vibration is the biggest impediment at present. Changing the proximity of the TMS coil to the magnet had minimal effect on the delay required to obtain clean EPI data. A bigger concern is the effect of TMS pulses on RF coil electronics, whether or not imaging artifacts are observed. As noted, we have had one unexplained coil failure when using TMS-fMRI and it is prudent to consider the potential effects for each TMS coil placement.

**Supplemental Acknowledgments**

The supplemental material was prepared primarily by BI and DS.

**Supplemental References**

1. Bohning, D., et al., *A combined TMS/fMRI study of intensity-dependent TMS over motor cortex.* Biological psychiatry, 1999. **45**(4): p. 385-394.

2. Bohning, D.E., et al., *BOLD‐fMRI response vs. transcranial magnetic stimulation (TMS) pulse‐train length: testing for linearity.* Journal of Magnetic Resonance Imaging: An Official Journal of the International Society for Magnetic Resonance in Medicine, 2003. **17**(3): p. 279-290.

3. BOHNING, D.E., et al., *Echoplanar BOLD fMRI of Brain Activation Induced by Concurrent Transcranial Magnetic Stimulation.* Investigative Radiology, 1998. **33**(6): p. 336-340.

4. Denslow, S., et al., *Cortical and subcortical brain effects of transcranial magnetic stimulation (TMS)-induced movement: an interleaved TMS/functional magnetic resonance imaging study.* Biological psychiatry, 2005. **57**(7): p. 752-760.

5. Bergmann, T.O., et al., *Concurrent TMS-fMRI for causal network perturbation and proof of target engagement.* NeuroImage, 2021. **237**: p. 118093.

6. Mizutani-Tiebel, Y., et al., *Concurrent TMS-fMRI: Technical Challenges, Developments, and Overview of Previous Studies.* Frontiers in Psychiatry, 2022. **13**.

7. Weiskopf, N., et al., *Image artifacts in concurrent transcranial magnetic stimulation (TMS) and fMRI caused by leakage currents: modeling and compensation.* Journal of Magnetic Resonance Imaging: An Official Journal of the International Society for Magnetic Resonance in Medicine, 2009. **29**(5): p. 1211-1217.

8. De Lara, L.I.N., et al. *Simulations of a birdcage coil B 1+ field on a human body model for designing a 3T multichannel TMS/MRI head coil array*. in *2018 40th Annual International Conference of the IEEE Engineering in Medicine and Biology Society (EMBC)*. 2018. IEEE.

9. Navarro de Lara, L.I., et al., *Evaluation of RF interactions between a 3T birdcage transmit coil and transcranial magnetic stimulation coils using a realistically shaped head phantom.* Magnetic resonance in medicine, 2020. **84**(2): p. 1061-1075.

10. Reese, T.G., et al., *Reduction of eddy‐current‐induced distortion in diffusion MRI using a twice‐refocused spin echo.* Magnetic Resonance in Medicine: An Official Journal of the International Society for Magnetic Resonance in Medicine, 2003. **49**(1): p. 177-182.
